# Genome-Wide Stochastic Adaptive DNA Amplification at Direct and Inverted DNA Repeats in the Parasite *Leishmania*

**DOI:** 10.1371/journal.pbio.1001868

**Published:** 2014-05-20

**Authors:** Jean-Michel Ubeda, Frédéric Raymond, Angana Mukherjee, Marie Plourde, Hélène Gingras, Gaétan Roy, Andréanne Lapointe, Philippe Leprohon, Barbara Papadopoulou, Jacques Corbeil, Marc Ouellette

**Affiliations:** Centre de Recherche en Infectiologie, Centre de Recherche du CHU de Québec, Québec, Canada; Baylor College of Medicine, United States of America

## Abstract

Gene amplification of specific loci has been described in all kingdoms of life. In the protozoan parasite *Leishmania*, the product of amplification is usually part of extrachromosomal circular or linear amplicons that are formed at the level of direct or inverted repeated sequences. A bioinformatics screen revealed that repeated sequences are widely distributed in the *Leishmania* genome and the repeats are chromosome-specific, conserved among species, and generally present in low copy number. Using sensitive PCR assays, we provide evidence that the *Leishmania* genome is continuously being rearranged at the level of these repeated sequences, which serve as a functional platform for constitutive and stochastic amplification (and deletion) of genomic segments in the population. This process is adaptive as the copy number of advantageous extrachromosomal circular or linear elements increases upon selective pressure and is reversible when selection is removed. We also provide mechanistic insights on the formation of circular and linear amplicons through RAD51 recombinase-dependent and -independent mechanisms, respectively. The whole genome of *Leishmania* is thus stochastically rearranged at the level of repeated sequences, and the selection of parasite subpopulations with changes in the copy number of specific loci is used as a strategy to respond to a changing environment.

## Abstract 2

The human parasite *Leishmania* uses adaptive gene rearrangements and amplification involving repeated sequences on a genome-wide scale as one strategy to adapt to a changing environment.

## Author Summary

Variations in the copy number of DNA segments account for a substantial amount of genome diversity of most organisms. DNA amplification, a contributor to copy number variation, can occur in response to various stresses or after altered growth conditions, leading to extensive and often reversible genetic variation. DNA amplification in the parasite *Leishmania* occurs outside the normal chromosomes and arises by DNA rearrangements involving homologous repeated sequences. We show here that such repeated sequences are widespread in the *Leishmania* genome and that most of the *Leishmania* genome is subject to stochastic gene rearrangements mediated by these low-copy repeat sequences. Thus, although cells in the population have a common core genome, many individual cells will differ from the rest of the population by carrying one or more distinct extrachromosomal amplicon. Upon selection with either drugs or culture conditions, a subpopulation can emerge where the amplicon copy number per cell increases, and this clone of cells can then expand to dominate the population. We propose that *Leishmania* uses adaptive gene amplification at a genome-wide scale as one strategy to adapt to a changing environment.

## Introduction

**Figure 1:**
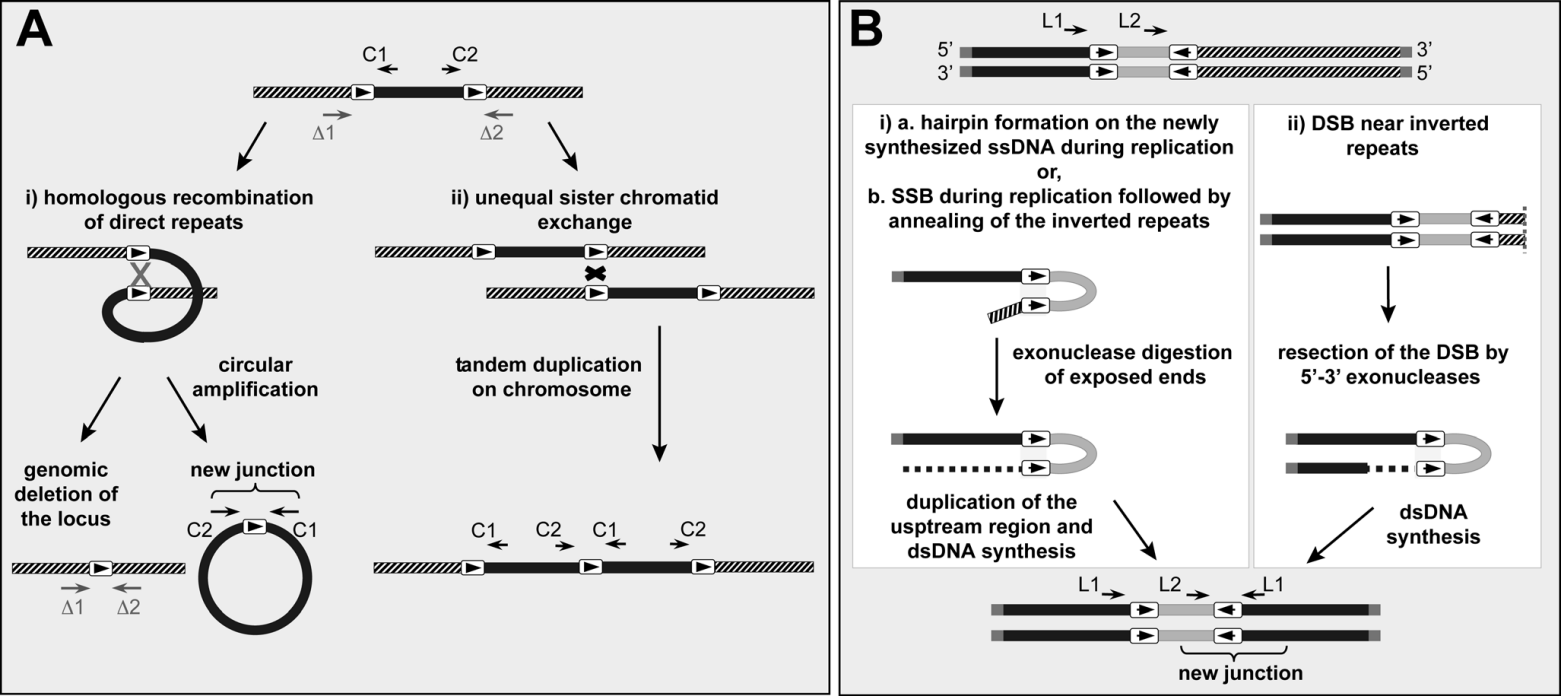
Models for gene amplification in *Leishmania*. (A) HR between DRs can lead to circular amplification (i) or to tandem duplication of the locus by nonequal crossing at sister chromatids (ii). The amplification can be nonconservative, leading to the deletion of the region amplified, or conservative (e.g., within replication forks), without genomic deletion. C1 and C2 are primers used to detect novel junctions formed by recombination between DRs. Arrows Δ1 and Δ2 are primers used to demonstrate a locus deletion between the DRs. (B) Role of IRs in the formation of linear amplicons. One model (i) suggests that IRs cause hairpin formation during replication and trigger the replication fork to stall and dissociate [7],[21]. The annealing of the repeats is used to prime DNA synthesis, leading to locus duplication up to the telomeric end [28]. Other mechanisms described in yeast and/or mammalian cells can also explain duplication events: (b) a single-strand break (SSB) during replication close to IRs or (ii) a double-strand break (DSB) initiating a linear duplication. The black bars represent the DNA segments that are amplified. L1 and L2 are primers used to detect novel junctions formed by rearrangements at IRs. The arrows in boxes indicate repeated sequences.

Copy number variations (CNVs) account for a substantial amount of genomic variability in mammalian genomes (reviewed in [1]). DNA amplification, a contributor of CNVs, has been reported in response to various stresses or after altered growth conditions, and can lead to extensive and often reversible genetic variations (reviewed in [2],[3]). Several models have been proposed to explain DNA amplification mechanisms [2]–[4]. Extrachromosomal circular DNAs can be the products of gene amplification in mammalian cells and in the protozoan parasite *Leishmania* (reviewed in [5],[6]). In *Leishmania*, DNA circles are generated by homologous recombination (HR) between direct repeated sequences (DRs) (Figure 1A) [7],[8]. DNA amplification can also lead to palindrome formation. Increasing evidence suggests that palindromes are initiated at the level of inverted repeats (IRs). Indeed IRs are known to increase chromosome instability during replication, leading to hairpin formation and representing a substantial source of DNA breakage and rearrangement (Figure 1B). IRs have been shown to initiate inverted duplications in yeast cells [9]–[11], in protozoa [4],[7],[8], and in mammalian cells [12].

*Leishmania* is an early diverging eukaryote whose genes are expressed constitutively as part of long polycistronic units where the RNAs are matured by coupled transsplicing and polyadenylation (reviewed in [13]) and by epigenetic marks [14]–[16]. Gene regulation occurs mostly at the posttranscriptional and (post)translational levels [13] with no control at the level of transcription initiation, in part due to the lack of several general transcription factors [17]. *Leishmania* display, however, additional strategies to modulate the expression of specific genes when selective pressure is applied. For example, *Leishmania* cells selected for resistance to cytotoxic compounds often amplify or delete a number of specific loci coding for either drug targets or drug transporters (reviewed in [6]). These amplified DNAs are generally extrachromosomal and found either as circular elements [18]–[21] or as linear minichromosomes [22]–[27]. These elements do not appear to have a specific origin of replication and are usually lost when drug pressure is removed [18],[28],[29], although some extrachromosomal elements are maintained in absence of drug pressure [22],[23],[25],[30].

The dihydrofolate reductase-thymidylate synthase gene *DHFR-TS* is amplified as part of extrachromosomal circles in *Leishmania* cells resistant to the antifolate methotrexate (MTX) [18],[28],[31]. In the few cases studied, these circles were formed by HR between DRs ranging in size, depending on the species, from 575 to 837 bp (Figure 1A) [28]. Similarly, the gene coding for the ABC protein MRPA can be amplified in *Leishmania* cells resistant to antimonials (SbIII), the first-line antileishmanial drug, by HR between DRs ranging in size from 198 to 1,389 bp (Figure 1A) [7],[29],[32]. In contrast, minichromosomes containing large inverted duplications [25],[26],[28],[30] are generated by the annealing of IRs [28] followed by duplications extending to telomeric ends (Figure 1B).

Repeated sequences used for DNA amplification are generally noncoding and are interestingly highly conserved between different *Leishmania* species [28]. These intergenic sequences may have been maintained to facilitate the amplification of key genomic loci essential to respond to changing growth conditions. Alternatively, such low-copy repeated sequences may be more abundant than previously thought and could be used by *Leishmania* as a platform to amplify several segments of its genome. We present here both bioinformatics and functional analyses revealing that homologous repeated sequences are widespread in the *Leishmania* genome and that most of the *Leishmania* genome is stochastically subjected to gene rearrangements at the level of these low-copy repeated sequences. Subpopulations of parasites with amplified DNA segments can be selected and we propose that *Leishmania* uses adaptive gene amplification at a genome-wide scale as one strategy to adapt to its changing environment.

## Results

### Genome-Wide Distribution of Repeated Sequences in *Leishmania*

**Figure 2:**
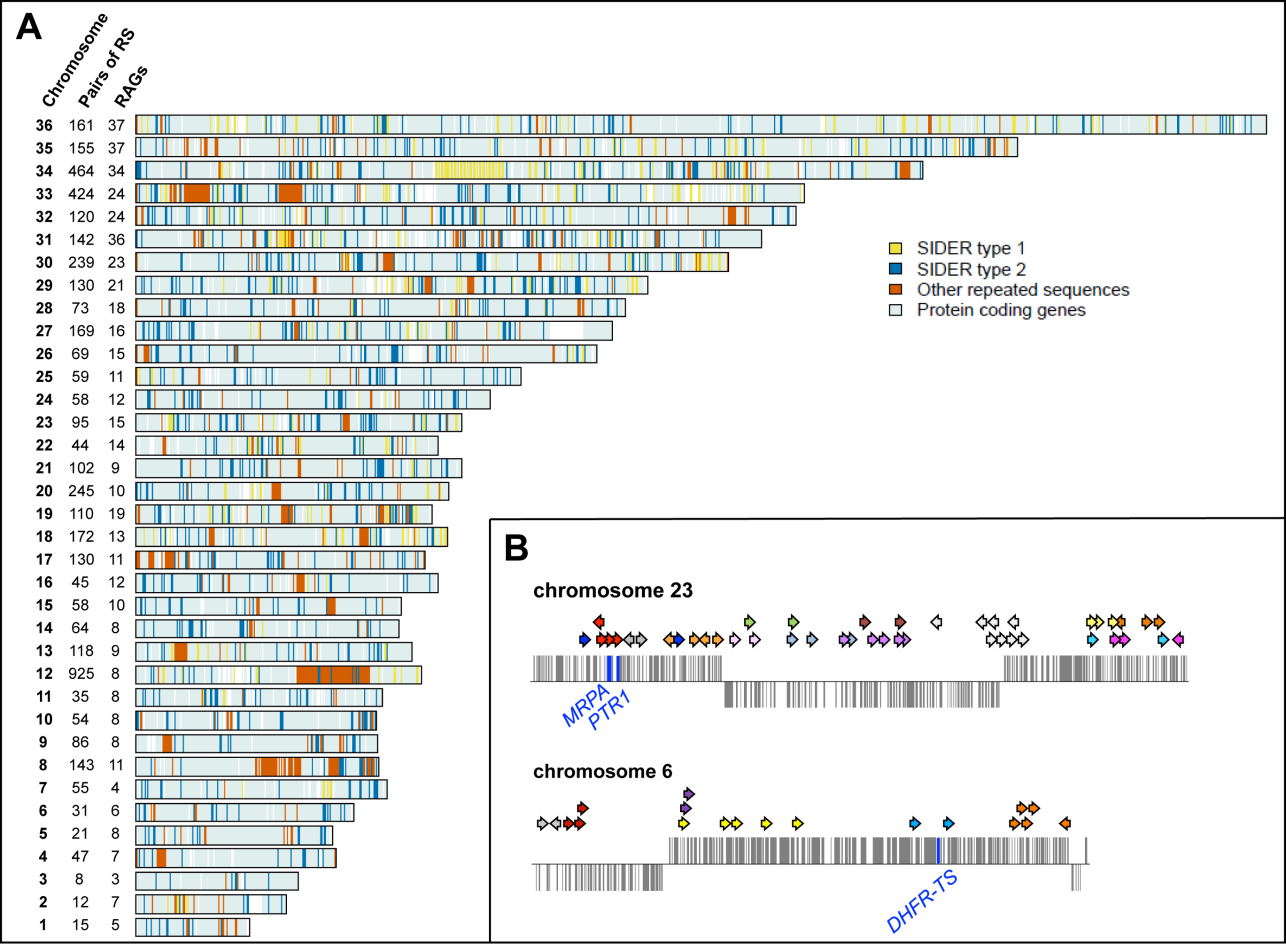
Genomic distribution of repeated sequences in the *L. major* genome. (A) The number of pairs of repeat sequences (RSs) is indicated for each of the 36 *L. major* chromosomes. The repeats are part of distinct RAGs. The repeats belonging to the SIDER retroposon family are the most abundant and are represented by yellow (SIDER1) or blue bars (SIDER2). Other repeated sequences are represented by orange bars. (B) Repeats on chromosomes 6 and 23 of *L. major*. Arrows indicate the orientation and the approximate location of the repeats. Repeats of the same RAG for each chromosome are displayed with the same color.

The noncoding DRs used for *DHFR-TS* amplification are highly conserved (86% identical) between *Leishmania major* and *Leishmania infantum* [28]. This interspecies conservation led us to hypothesize that either specific loci are subjected to considerable CNVs or alternatively that the *Leishmania* genome has more repeated sequences than initially anticipated. Indeed, the published analyses of the genome sequences of several *Leishmania* species did not reveal the presence of extensive repeated sequences [17],[33]. The genome sequence of *L. major* Friedlin was screened for noncoding repeated sequences. To this end, intergenic regions were aligned to their respective entire chromosome sequences using blastn. Repeated sequences were filtered for lengths between 0.2 and 2.5 kbp with a minimum identity of 85%, as a high level of homology is required for recombination in *Leishmania* [34]. There is no evidence for non-HR in *Leishmania* and HR is likely to require up to 95% of homologous sequences [35]. According to these criteria, we identified 1,926 repeats in the *L. major* genome (Figure 2A) for which we assigned a unique identifier (Tables S1 and S2). These repeats represent 5% of the *Leishmania* genome and are scattered throughout all the chromosomes and are either in a direct or in an inverted orientation, as illustrated for chromosomes 6 and 23 in Figure 2B.

We clustered the repeated sequences into Repeat Alignment Groups (RAGs), each RAG being composed of all the members of a same repeat family (with 85% minimum identity). Using these criteria, we assembled 507 RAGs in the *L. major* genome by BLAST. Most RAGs, 490 out of 507, have fewer than 10 members, with 242 RAGs consisting of only two unique repeated sequences (Tables S1 and S2). RAGs are usually confined to one specific chromosome, with two exceptions, RAG17 with its 33 repeats distributed on 13 chromosomes and RAG418 with its 29 repeats present on two chromosomes (Tables S1 and S2).

**Figure 3:**
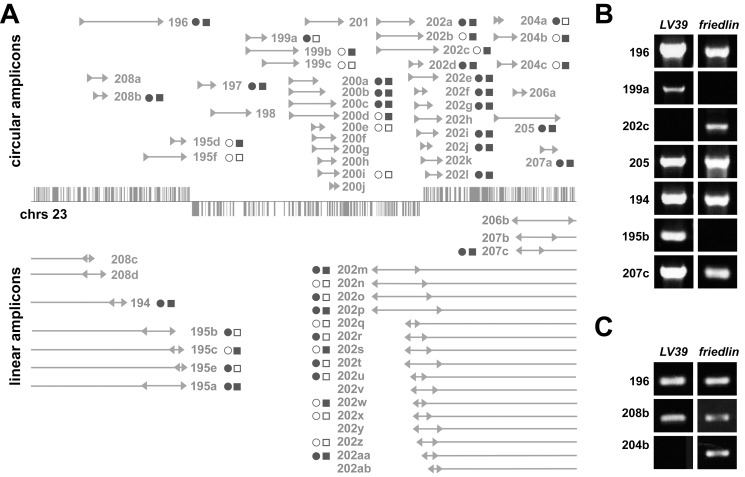
DNA amplification/deletion occurs naturally in *Leishmania* parasites. (A) Chromosome 23 with its three large polycistronic units is shown. Each vertical bar represents a gene. Intergenic regions of chromosome 23 were scanned for repeated sequences, and 15 RAGs (194–208) theoretically leading to 95 amplicons were detected (Table S1). RAG 203 (and its 29 putative amplicons) is not represented because it corresponds to complex duplicated regions difficult to investigate by PCR. We tested for the presence of 48 amplicons by PCR. PCR fragments of the expected size provided evidence for 25 amplicons formed at the level of DRs (upper map) and 15 amplicons formed at the level of IRs (lower map). PCR products of the right size detected (filled circle and filled square) or not detected (open circle and open square) in *L. major* LV39 (circles) and Friedlin (squares), respectively. (B) Selected examples of the detection of PCR products compatible with DNA rearrangements mediated at the level of either DRs or IRs in *L. major* strains LV39 or Friedlin. (C) Detection of PCR products diagnostic of deletion of three loci at the level of DRs using primers equivalent to Δ1 and Δ2 of Figure 1A.

Within several of the 507 RAGs, we detected the presence of sequences part of short interspersed degenerate retroposons (SIDERs), which are distributed in the *Leishmania* genome [36],[37] and proven to regulate gene expression at both the posttranscriptional (SIDER2) or translational (SIDER1) level [36],[38]. SIDERs are degenerate and thus do not fall into a single RAG but are included in several of the RAGs (Table S1). We found 359 SIDER1 repeats and 948 SIDER2 repeats, accounting for 67.9% of the 1,926 repeated sequences of *L. major*. Because several RAGs have more than one pair of repeats per chromosome (e.g., see Figure 2B and Table S1), we estimated that the 1,926 repeats in the 507 RAGs can lead to 4,601 potential amplicons in *L. major* (Table S2). The distances between DRs (Figure S1A) or IRs (Figure S1B) are on average between 1 and 100 kb. IRs are found in general closer to telomeres, whereas DRs appear more evenly distributed along the chromosomes (Figure S1C and S1D; see also Figures 2B and 3A).

Similar bioinformatics analyses were performed on the *L. infantum* and *L. braziliensis* genomes. Blastn screens revealed a total of 1,886 repeats in *L. infantum* and 2,058 in *L. braziliensis* that were assembled, respectively, into 513 and 619 RAGs, and could potentially lead to 3,165 and 3,183 amplicons (Tables S3 and S4). *L. major* and *L. infantum* share over 60% of their RAGs with a strong conservation in synteny. *L. braziliensis*, a representative of the *Viannia* subgenus, is distant from the two other species, and with the search criteria at 85% identity, only 10% and 16% of RAGs were conserved within *L. major* and *L. infantum*, respectively.

### Stochastic Genome-Wide Gene Amplification in *Leishmania*

We have shown previously that PCR allows the detection of novel junctions formed after genomic rearrangement triggering circular (C1 and C2 primers of Figure 1A) or linear (primers L2 and L1 in Figure 1B) amplifications in drug-resistant *Leishmania* cells [28],[29]. We also observed the amplification of a *GSH1* locus in a wild-type background by using a PCR assay with higher sensitivity [39]. We thus tested whether other rearrangements could be detected in wild-type cells using similar sensitive PCR assays. Chromosome 23 contains a minimum of 15 RAGs (RAG194–RAG208), with a total of 54 repeats that could theoretically lead to the formation of 95 amplicons. We tested the rearrangement of 48 of these amplicons and detected a PCR product of the expected size for 40 of them. Twenty-five amplicons were produced by HR between DRs and 15 after annealing of IRs (Figure 3A). For example, RAG195 includes four repeats in direct and inverted orientations (in orange in Figure 2B). Out of the six potential amplicons, five were detected in at least one of the two strains, the amplicon 195d was generated by HR between DRs, whereas the amplicons 195a,b,c,e are consistent with a rearrangement at IRs (Figure 3A,B). Selected PCR products were sequenced, and these rearrangements were confirmed.

We were able to consistently detect 60% to 80% of the predicted amplicons in any given population of late-log phase cultures for two strains of *L. major*, LV39 and Friedlin (see for examples Figure 3A and 3B). A similar frequency of rearrangements was observed in *L. infantum* JPCM5 and in our *L. infantum* lab strain 263. Although most work was conducted with promastigotes, the amplicons could also be detected in intracellular *Leishmania* inside macrophages (Figure S2). These genomic rearrangements in unselected strains are both global and stochastic, as the sets of amplicons are not always identical (Figure 3A,B). Through dilution experiments and using the *L. major* MTX60.4 line with amplification of the DHFR-TS locus, we estimated the frequency of loci rearrangements to be approximately 10^−6^ to 10^−7^/cell (see Materials and Methods and Figure S3).

Gene amplification in *Leishmania* can be either conservative, where the genomic region amplified remains intact, or nonconservative, where the chromosomal locus amplified is deleted (Figure 1A). Conservative amplification is observed more often in highly drug-resistant mutants, most likely because deletion of large genomic regions is associated with a cost, but nonconservative amplification has been observed previously with arsenite- [32] and MTX-resistant mutants [28]. We thus tested whether locus deletion was detectable in populations using appropriate primers (the equivalent of Δ1 and Δ2 in Figure 1A). We tested for the deletion of the chromosomal segments between the DRs of the RAGs 196, 208b, and 204b, and obtained PCR products of the appropriate size and sequence consistent with a locus deletion (Figure 3C).

### Adaptive Gene Amplification in *Leishmania*

**Figure 4:**
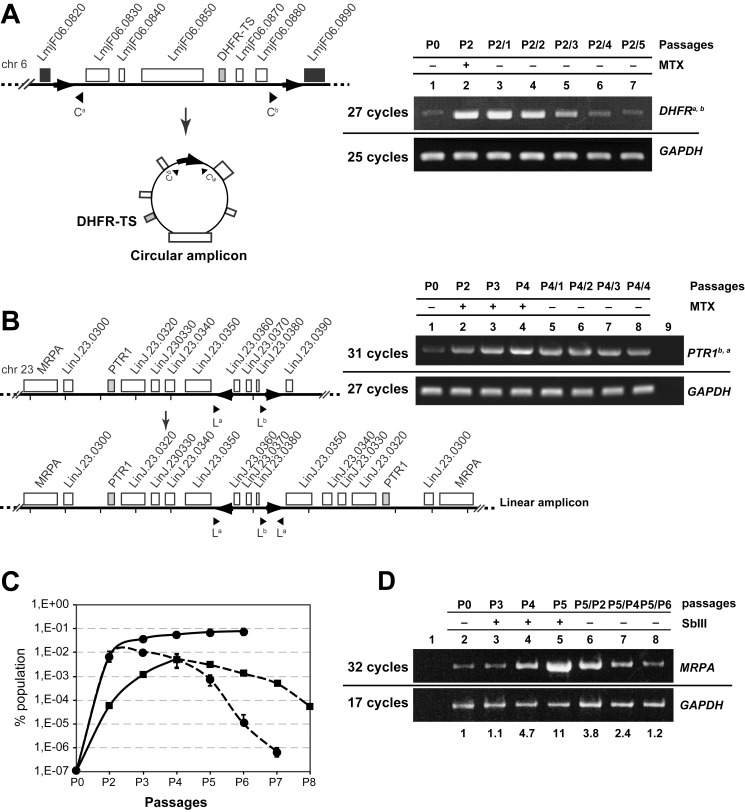
Adaptive gene amplification upon drug selection. (A) Schematic representation of the *DHFR-TS* locus and the circular amplicon generated by HR between DRs (black arrows) on chromosome 6 in *L. major*. Genomic DNA was extracted from WT and MTX stressed promastigotes. Amplicon detection in the unstressed WT population (right panel, lane 1), after two passages with MTX (at EC_50_, 0.2 µM) (right panel, lane 2), and after removing MTX pressure (lanes 3–7). C^a^ and C^b^ are the primers used for detecting the rearrangements. (B) Schematic representation of the *PTR1* and *MRPA* locus and the linear amplicon with inverted duplications generated after annealing of IRs (black arrows) on chromosome 23 in *L. infantum* (left panel). Detection of the *PTR1* amplicons by PCR using primers L^a^ and L^b^ in the unstressed WT 263 population (right panel, lane 1), after four passages with MTX (at EC_50_, 0.2 µM) (lanes 2–4), and after removing MTX pressure (lanes 5–8). Lane 9 is a no DNA template control. (C) Kinetics of the selection and loss of the *DHFR* circular amplicon containing cells in LV39 (filled circle) and *PTR1* linear amplicon containing cells in *L. infantum* (filled square) under MTX pressure (continuous line) and after drug removal (dashed lines). Average of three biological independent experiments is shown. (D) Adaptive *MRPA* gene amplification upon SbIII selection. Semiquantitative PCR was performed with primers L^a^ and L^b^. Genomic DNA was extracted from *L. infantum* WT (lane 2) cells stressed with SbIII at 160 µM (4×EC_50_) (lanes 3–5) and from cultures after drug removal (lanes 6–8). Lane 1 is a no DNA template control. Densitometric ratios of PCR band intensities are indicated at the bottom. One representative experiment out of four is shown. Amplification of the chromosomal *GAPDH* gene was used as a reference to normalize the amount of template DNA loaded.

We hypothesized that cells carrying advantageous preexisting amplifications could be positively selected to eventually represent a larger proportion of the population. Additionally, amplicons should be lost when selection is removed, confirming that adaptive gene amplification is reversible. We therefore exposed *L. major* LV39 and *L. infantum* 263 to MTX pressure, a drug known to select either for *DHFR-TS* or *PTR1* amplification in *Leishmania* [18],[24],[31]. Using a semiquantitative PCR approach with appropriate primers (Figure 4A), we detected the *DHFR-TS* circular amplicon in the untreated wild type *L. major* LV39 population (Figure 4A, lane 1) at an estimated frequency of 10^−6^ to 10^−7^/cells (Figure S3). After only two passages (∼18 generations) with 0.2 µM MTX (the EC_50_ value), we estimated that approximately 1% of the population carried the *DHFR-TS* amplicon (Figure 4A, lane 2), and after six passages with 0.2 µM MTX, this proportion increased to 10% of the population (Figure 4C). Inversely, when the drug pressure was removed, the estimated proportion of the cells containing the *DHFR-TS* amplicon decreased rapidly (Figure 4A,C). In the unstressed wild-type *L. infantum* 263 population, the *DHFR-TS* circular amplicon could not be detected; hence, no positive selection could be observed after MTX pressure. Instead, we detected the rearrangement resulting in a *PTR1* linear amplicon in the unstressed *L. infantum* 263 population (Figure 4B) and monitored its positive selection under 0.2 µM MTX pressure. The proportion of the *PTR1* linear amplicon-containing cells increased to reach 1% of the population after four passages with the drug and again decreased when the selection was removed, albeit less rapidly than for *DHFR-TS* circular amplicons (Figure 4B,C).

We also analyzed the adaptive gene amplification in *L. infantum* 263 population selected with SbIII, for which amplification of the *MRPA* gene has been previously reported [40]. The *MRPA* gene is located near *PTR1* and can be amplified as part of a linear amplicon mediated by rearrangements at the level of the same IRs as for *PTR1* (Figure 4B). We indeed observed a gradual increase in the proportion of the *MRPA* linear amplicon-containing cells in the population following five passages with 160 µM of SbIII (Figure 4D, lanes 3–5). When the SbIII stress was removed (Figure 4D, lanes 6–8), we observed a decrease of the PCR product indicative of the loss of the *MRPA* linear amplicon in the population.

**Figure 5:**
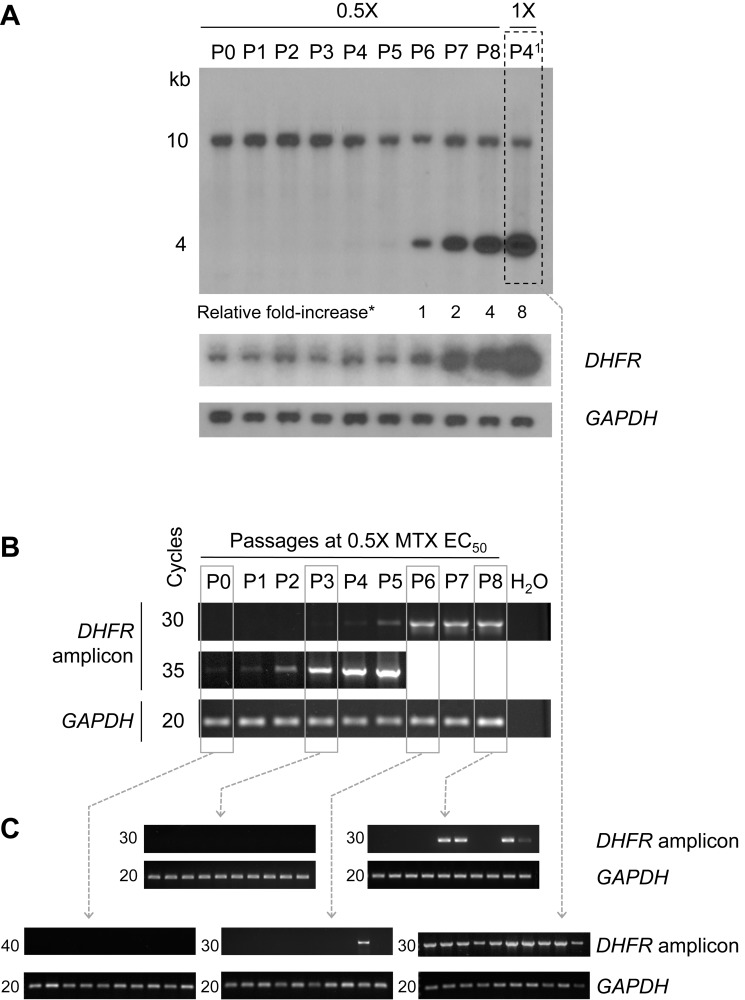
Early events of gene amplification in *Leishmania* and expansion of clones with amplified DNA upon selection. *L. major* was either cultured in the presence of 0.5× MTX EC_50_ for eight continuous passages (P1–P8) or cultured for one passage at 0.5×EC_50_ and then shifted for four passages at 1× MTX EC_50_ (P4^1^) (see Figure S4A for the selection scheme). (A) DNAs extracted from the population of parasites grown at 0.5×EC_50_ MTX for up to eight passages (P1–P8) and for four passages at 1×EC_50_ MTX (P4^1^) were analyzed by Southern blot for the presence of *DHFR-TS* circles. The blot was hybridized to a probe corresponding to the first 1,000 bp of LinJ.06.0830 that discriminate the chromosomal loci at 10 kb from its amplified region at 4 kb (see Figure S4B). The blot was also hybridized with a probe covering the coding sequence of *DHFR-TS* (*DHFR*) and to a *GAPDH* probe to monitor the DNA loaded in each lane. Fold increases were normalized with the *GAPDH* signal. (B) DNAs extracted from the population of parasites at every 0.5×EC_50_ passages (P1–P8) were further analyzed by semiquantitative PCR for the presence of the new junction derived from HR between the direct repeats flanking the *DHFR-TS* locus (see Figure 4A). The number of PCR cycles for the *DHFR-TS* amplicon and the *GAPDH* control is indicated. For passages P6–P8, the PCR signal was saturated at cycle 35 and is thus not shown. (C) Parasites from five selected passages (boxed in A and B) were cloned on plates. For each condition, total DNA was extracted from 10 randomly selected colonies and tested for the presence of *DHFR-TS* amplification by PCR. The number of PCR cycles for the *DHFR-TS* amplicon and the *GAPDH* control is indicated on the left of each panel.

The increased intensities observed by PCR upon selection possibly reflect a combination of both the successive divisions of the amplicon-containing cells but also of an increased copy number of these amplicons in a given number of cells. To test this, we selected *L. major* for MTX resistance both by keeping cells at 0.5×EC_50_ for up to eight passages but also by incremental increase of the drug (Figure S4A). Amplification of the *DHFR-TS* locus was tested by both Southern blot analyses (Figure 5A) and by PCR (Figure 5B). An EcoRV-BglII digest was hybridized to a probe that can discriminate the 10 kb chromosomal locus and the 4 kb rearranged circle's band (Figure S4B). In cells grown without MTX (P0) and cells grown in the presence of 0.5×EC_50_ MTX for up to four passages (P4), we observed the 10 kb chromosomal band but not the 4 kb band diagnostic of the circle (Figure S4B, Figure 5A). However, at P5 (faintly) and at passages 6, 7, and 8, we see a gradual increase hybridization intensity of the 4 kb band (Figure 5A). A paralleled and expected increase was also observed when the blot was hybridized with a *DHFR-TS* probe, whereas the signal with the control *GAPDH* probe remained constant (Figure 5A). Selection with higher drug concentration enriched more rapidly for more circles [see P4 at 1×EC_50_ (P4^1^), Figure 5A], although at one point a plateau is reached in terms of amplified circles, as an average of 20 copies of *DHFR-TS* was observed in comparison to control probes at either 2× or 8×EC_50_ (Figure S4C). The next step was to test whether these amplicons are evenly distributed in each cell within the population. At P6, the 4 kb rearranged band is of similar intensity than the chromosomal band (Figure 5A), whereas at P8 and P4^1^ the hybridization ratio between the rearranged bands and the chromosomal bands (or between *DHFR-TS* and *GAPDH*) are 4 and 8, respectively (Figure 5A). This suggests that in P6, P8, and P4^1^, there is an average of one, four, and eight circles per cell, although this could also be unevenly distributed. To test this, we complemented the Southern blots and the semiquantitative PCR with a colony-based PCR assay. We plated cultures of P0, P3, P6, P8, and P4^1^, and 10 colonies for each population culture were immediately processed and tested directly by PCR for the rearranged fragment diagnostic of the *DHFR-TS* locus amplification. Using this qualitative colony-based PCR approach, we could not detect PCR products in any of the clones derived from P0 and P3. A PCR fragment diagnostic of the *DHFR-TS* amplicon was observed in 1 out of 10 clones derived from P6 (Figure 5C), suggesting that not all cells within the population had the amplicon, but several cells had many copies of the amplicon to provide the hybridization signals observed in Figure 5A. Similarly, when P8 with its average of four circles per cell (Figure 5A) was cloned, only four out of 10 colonies had the amplicon; thus, several cells must have more than four copies to lead to the hybridization signals shown in Figure 5A. The average number of circles per cell in P4^1^ was determined to be eight (Figure 5A), and all 10 clones derived from P4^1^ had the amplicon, suggesting that the copy number of the amplicon must be in average eight to explain the hybridization results of Figure 5A. However, there could be variation in the copy number in individual cells of the population (as our assay in Figure 5C cannot discriminate for this). When the selection is pursued, the copy number of the amplified locus further increases to an average of 20 (Figure S4C). The results of Figure 5 support the concept that few cells within the population have initially the rearranged structure (P0 to P5 in Figure 5A and 5B). Selective pressure will lead to an increase in the proportion of the rearranged structure within the population (Figure 5). The experiment of Figure 5C is showing that an increased number of cells within the population is emerging with the amplicon, and experiments of Figure 5A and Figure S4C are showing that upon selection there is also an increase in the copy number of amplicons within the cells.

**Figure 6:**
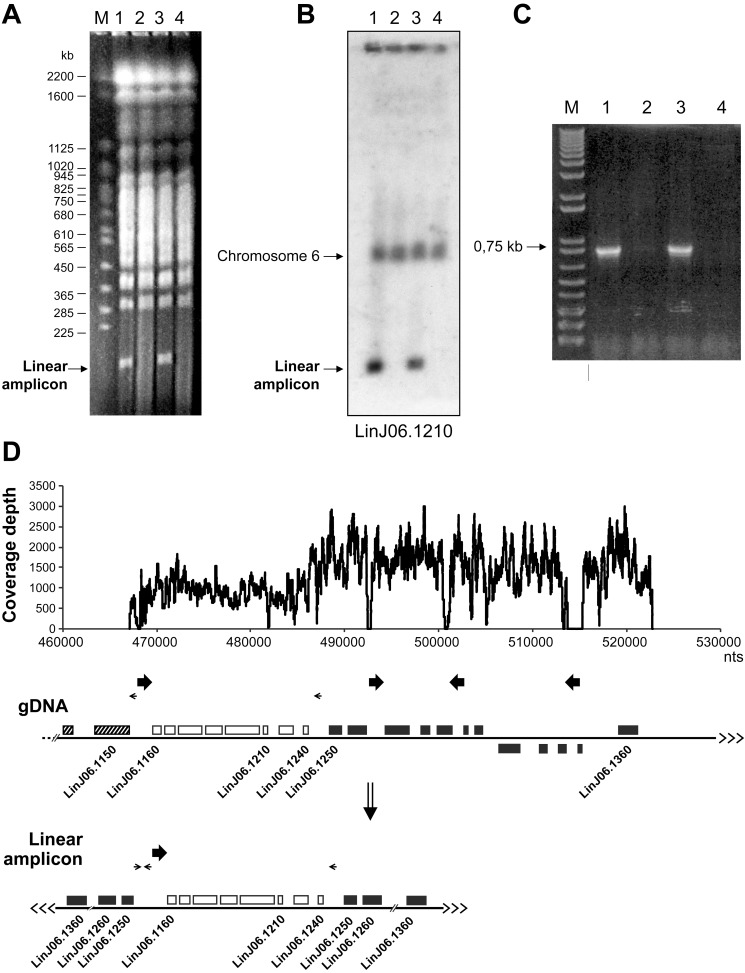
CNV of a linear amplicon derived from chromosome 6 in the presence of fetal bovine serum. (A) The small chromosomes of *L. infantum* were separated by pulse-field gel electrophoresis. Total DNA was isolated from cells cultured with FBS (lane 1) and from cells cultured in serum-free medium for 15 passages (lane 2), FBS was then added for 15 passages (lane 3), and removed again for 15 passages (lane 4). Lane M, marker of *S. cerevisiae* chromosomes. (B) Southern blot hybridized to a probe derived from *LinJ06.1210*. (C) PCR assay mapping the rearrangement leading to the linear amplicon shown in (D). The primers used are indicated by small arrows located between *LinJ06.1150* and *LinJ06.1160* and *LinJ06.1240* and *LinJ06.1250*. A line indicates nonadjacent lanes that have been brought together for producing the figure. (D) Sequence coverage depth as determined by NGS of the linear amplicon. Below are the genomic organization of the subtelomeric region of chromosome 6 in *L. infantum* and the structure of the linear amplicon with its inverted duplication. Large arrows indicate the presence of repeated sequences where one of the rearrangements leading to extrachromosomal linear amplification occurred, whereas thin arrows indicate the location of PCR primers.

Gene amplification unrelated to drug selection has also been reported in *Leishmania* [41]. Interestingly, pulse-field gels revealed the presence of an amplified linear DNA in our *L. infantum* 263 strain grown *in vitro* for several years (Figure 6A, lane 1). We discovered that the selection of this linear amplicon is linked to the supplementation of FBS to the medium. Indeed, the minichromosome is positively selected when FBS is added to the medium (Figure 6A, lanes 1 and 3), whereas removing the FBS leads to its gradual loss (Figure 6A, lanes 2 and 4). Selective loss or gain of these amplicons was most clearly observed after 15 passages. Next generation sequencing (NGS) of the purified minichromosome using Illumina paired-end reads revealed that it is derived from chromosome 6 (Figure 6D), which was confirmed by Southern blot hybridization (Figure 6B). The amplicon extended from a region between *LinJ06.1150* and *LinJ06.1160* to one telomeric end of the chromosome (Figure 6D). Interestingly, a sequence that was repeated several times on chromosome 6 either in a direct or inverted fashion was found at one breakpoint between *LinJ06.1150* and *LinJ06.1160* (Figure 6D). Sequence coverage was consistent with a rearrangement between *LinJ06.1150* and *LinJ06.1160* and between *LinJ06.1240* and *LinJ06.1250*, although no obvious repeated sequences were apparent in the latter region (Figure 6D). A PCR assay (Figure 6C, lane 1) confirmed that this rearrangement occurred at the position suggested by NGS analysis (see the primers used in Figure 6D). The PCR fragment was only observed in cells with the linear amplicon (Figure 6C). The absence of IRs at the rearrangement that would have explained the formation of this linear amplicon may be due to secondary rearrangements due to the long culture history of the strain. This is consistent with the primers failing to detect amplification in cells without the amplicon (Figure 6C, lanes 2 and 4), as there are no continuous rearrangements that can take place. There must nonetheless be a low number of cells remaining with this amplicon, and this may explain the slow re-emergence (15 passages) of cells with amplicons upon growth with FBS. In future studies, it would be of interest to link the gene within the amplicon and the substrate within FBS driving the selection.

### Formation of Extrachromosomal Circles But Not of Linear Amplicons Is Facilitated by the *RAD51* Recombinase

**Figure 7:**
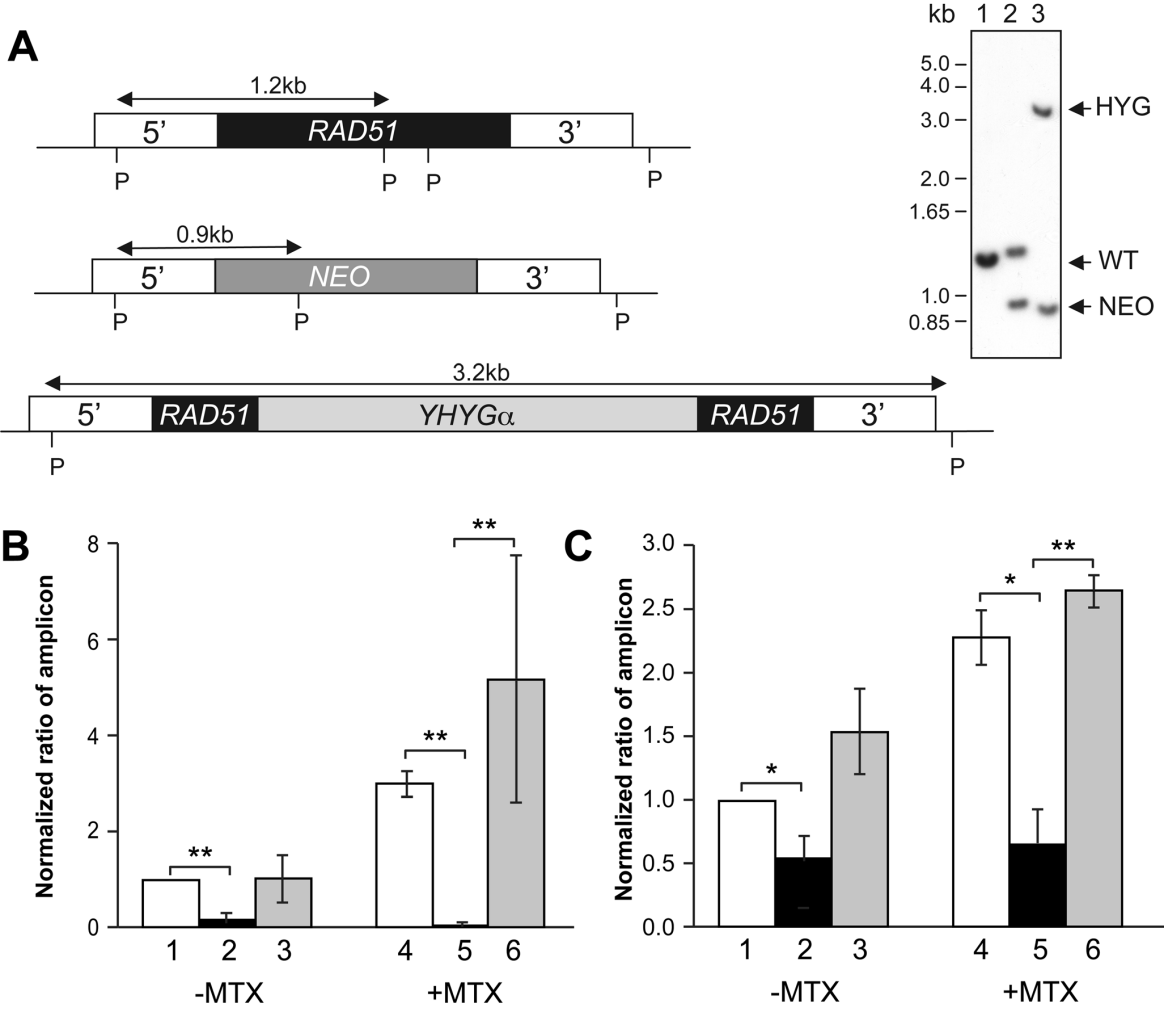
Adaptive gene amplification in *RAD51* ^−/−^ parasites. (A) Diagram of the *RAD51* locus in *L. infantum* WT with the *NEO* and *HYG* disruption cassettes (left panel). Southern blot analysis (right panel) of WT (lane 1); *RAD51/RAD51::NEO* (lane 2) and *RAD51::NEO/RAD51::HYG* (lane 3) genomic DNAs digested with PvuII (P) and hybridized with the 5′ UTR *RAD51* probe. (B and C) Circular amplicon selection in *RAD51* ^−/−^ parasites. *L. infantum* WT pSPα*ZEO*α (1 and 4), *RAD51* ^−/−^ pSPα*ZEO*α (2 and 5), and *RAD51* ^−/−^ pSPα*ZEO*α*RAD51* (3 and 6) were either cultured in the absence of drug (−) or in the presence of 0.2 µM MTX for five passages. Total DNA was extracted from cells, and semiquantitative PCRs were performed to detect *DHFR-TS* (B) or *PTR1* (C) circular amplicons. Amplification of the chromosomal *GAPDH* gene was used as reference to normalize the amount of template DNA. The data shown are averages of three independent experiments (*p≤0.05; **p≤0.005; ***p≤0.0005, two-tailed Student's *t* test).

We hypothesized that RAD51, the main recombinase in eukaryotes, would have an important role in the formation of circular amplicons, where a crossover is necessary between two homologous DRs (Figure 1A). The gene *LinJ.28.0580* encodes the closest homolog of RAD51 in *Leishmania*, sharing 70% homology with the yeast RAD51 protein. To test its role in HR and extrachromosomal circular amplification, we generated a *L. infantum RAD51* null mutant. A PvuII digest of *L. infantum* genomic DNA hybridized to a *RAD51* 5′flank probe should lead to a 1.2 kb fragment in WT (Figure 7A, lane 1). Integration of *NEO* and *HYG* expression cassettes should lead to 0.9 and 3.2 kb fragments, respectively, and the integrations were verified by Southern blots (Figure 7A). Moreover, no copy of *RAD51* was detected in the null mutant by PCR (Figure S5A,B). As expected, the *RAD51* null mutant was sensitive to the double-strand break inducing agent methyl methanesulfonate (Figure S5C), and the phenotype could be reversed by transfecting the null mutant with an episomal vector harboring the *RAD51* gene (Figure S5C). We investigated whether the *Leishmania RAD51* null mutant exhibited a decreased efficiency of HR by double crossover using a *BLA* gene replacement cassette tested previously to inactivate the *GSH1* gene [39]. Transfection of this cassette showed that the *RAD51* null mutant had a decreased ability to yield BLA-resistant colonies in comparison to wild-type cells or to the add-back revertant (Figure S5D).

The stochastic and adaptive gene amplification abilities of the *RAD51* null mutant were analyzed. The *DHFR-TS* circular amplicon was detected in *L. infantum* wild-type cells, but its level was significantly reduced in the unselected *RAD51* null mutant (Figure 7B, lanes 1 and 2), whereas wild-type level was observed in the add-back revertant (Figure 7B, lane 3). Following MTX pressure, an increased level of *DHFR-TS* circular amplicons was observed in the wild-type cells but not in the RAD51^−/−^ mutant cells (Figure 7B, lanes 4 and 5). Similar observations of reduced circle formation in the RAD51^−/−^ mutant were obtained for a *PTR1* circular amplicon (Figure 7C, lanes 2 and 5). The phenotype was reverted in the *RAD51* add-back rescued cells (Figure 7B,C, lanes 3 and 6).

**Figure 8:**
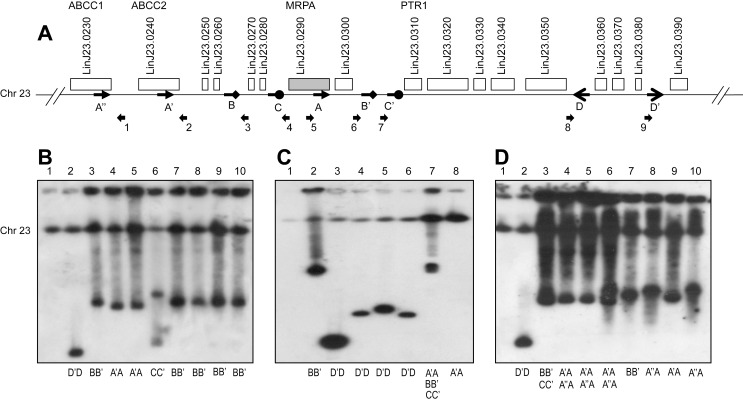
*MRPA* locus amplification upon arsenite selection and the role of the *RAD51* recombinase. (A) The *MRPA* locus and the repeats allowing its amplifications are shown; DRs are A–A′, A–A″, B–B′, and C–C′ and IRs are D–D′. Primers 1–9 were used to determine the rearrangements that occurred in *L. infantum* WT cells (B), in *RAD51* ^−/−^ (C), and in *RAD51* add-back revertant parasites (D) all selected for arsenite resistance (up to 250 µM = 5×EC_50_). The DNA of resistant clones was separated by pulse-field gel electrophoresis and hybridized to a *MRPA* probe. The rearrangement point for each amplicon was determined by PCR (Figure S7) and is indicated below for each lane. Lane 1, unselected population; lanes 2–10, independent arsenite-resistant clones.

Further evidence for the role of RAD51 in circular amplification was obtained by selecting wild-type and *RAD51* null mutant cells for arsenite resistance, a metal related to SbIII and a potent selector of circular *MRPA* gene amplification [7],[42]. Seven to nine independent clones of wild-type *L. infantum*, *RAD51* null mutant, and *RAD51* add-back revertant lines were obtained after *in vitro* step-by-step selection for high arsenite resistance. We analyzed the clones for *MRPA* gene amplification with a *MRPA* probe hybridized to pulse-field gels. Among the nine resistant clones derived from the wild-type background, eight had *MRPA* circles (Figure 8B, lanes 3–10) and one exhibited a *MRPA* containing linear amplicon (Figure 8B, lane 2) as deduced by their characteristic hybridization patterns. The extent of gene amplification was estimated in selected mutants by standard Southern blots (Figure S6). Seven resistant clones were obtained with the *RAD51* null mutant, of which only two clones harbored *MRPA* circular amplicons (Figure 8C, lanes 2 and 7). Four other clones presented *MRPA* linear amplifications (Figure 8C, lanes 3–6) and the remaining clone did not exhibit any extrachromosomal MRPA amplification, although hybridization at the chromosomal locus was more intense (Figure 8C, lane 8). A statistical analysis demonstrated that significantly fewer circles were observed (*p*<0.05) in the *RAD51* null mutant compared to wild-type cells. This is RAD51-specific, as *MRPA* coding circles were observed in the *RAD51* add-back rescue cells selected for arsenite resistance (Figure 8D).

Two different pairs of DRs can promote circular amplification of the *MRPA* gene (B–B′ and C–C′ in Figure 8A). Moreover, the *ABCC1*, *ABCC2*, and *MRPA* genes encode three members of the ABCC family of transporter proteins. They share a nucleotide binding site with high homology and these conserved sequences, although coding can be the substrate for HR and can thus support circular amplification (A, A′, and A″ in Figure 8A). PCR analyses of the arsenite-resistant clones containing *MRPA* circular amplicons revealed different *MRPA* circles depending on the pair of DRs used for rearrangement, and *MRPA* circular amplicons were formed and selected at the level of all of these repeats, although only one type of circle is usually found in one clone (Figures 8B and S7). The *MRPA* linear amplicons were all generated after annealing of the IRs D–D′ despite their size differences (Figure 8B, lane 2; Figure 8C, lanes 3–6; and Figure S7). This difference could be explained by secondary internal rearrangements and/or differences in telomeric sequences. In the *RAD51* add-back revertant clones resistant to arsenite, we observed a higher level of amplicons as deduced from the hybridization signals and a higher diversity of *MRPA* circles, as four clones exhibited at least two circular amplicons with different rearrangement points (Figure 8D, lanes 3–6 and Figure S7). Intriguingly, one *RAD51* null mutant clone (Figure 8C, lane 7 and Figure S7B) had evidence for at least three amplicons with different rearrangements. The *RAD51* null mutant resistant to arsenite with a stronger hybridization at the chromosomal locus had a positive PCR reaction between repeats A–A′ (Figure 8C, lane 8), suggesting a tandem intrachromosomal duplication (see Figure 1A).

## Discussion

Bioinformatics analyses indicated that repeated sequences are spread throughout the *Leishmania* genome (Figure 2, Tables S1–S4). Our screen was focused on noncoding sequences of at least 200 bp. We used this cutoff as HR in *Leishmania* was found to be more efficient with regions of homology of at least 200 bp [34]. However, shorter repeats or repeated coding sequences (see Figure 8) could also be used for genomic rearrangements. RAGs are usually in low copy, but many contain the extinct SIDER retroposons. These SIDER retroposons are too degenerated to be included into one RAG, but they appear to have a dual role: one structural, allowing DNA rearrangements, and a functional role, as SIDER-1 and -2 were found to modulate gene expression at the level of translation and mRNA stability, respectively [36],[38].

At the genome scale, these repeated sequences provide a platform for gene rearrangements. This was illustrated for chromosome 23, where we obtained experimental evidence that the entire length of the chromosome is subjected to rearrangements in unselected wild-type cells (Figure 3). These genome-wide rearrangements led to either circular or linear extrachromosomal elements, depending on whether rearrangements occurred at the level of DRs or IRs, respectively. Because the extrachromosomal nature of the amplified DNA cannot be tested in wild-type cells, we subjected cells to growth conditions that allowed the detection of amplification by nonsensitive techniques (such as in Figures 5A, 6, or 8). These were invariably extrachromosomal (reviewed in [6]). One exception is shown in Figure 8C (lane 7), suggesting a tandem intrachromosomal duplication event. Thus, we cannot exclude that some of the PCR products observed in unselected lines could be due to intrachromosomal tandem duplication (Figure 1A), but we favor the model of extrachromosomal amplification because experimental evidence for the latter abound in *Leishmania*. Our data suggest that extrachromosomal elements are initially present in a limited number of cells. Upon division and selection, the copy number of these elements increases, possibly by unequal segregation during cell division, as suggested for double-minute chromosomes (reviewed [43]). Cells with higher copy of the extrachromosomal elements have a selective advantage and can expand to become a majority of the population during further selection (Figure 5C).

DNA replication in *Leishmania* is not well understood but because plasmids with no *Leishmania* DNA sequences can replicate [44], it does suggest permissiveness. The fact that amplified DNAs are usually lost when selection is removed (Figures 4, 6, and 7) implies that they do not encode the sequences necessary to be maintained stably. The apparent permissiveness of *Leishmania* in DNA replication may explain why amplification can be observed so frequently. The phenomenon of stochastic gene amplification may also happen in other organisms, but this may not have been observed because these extrachromosomal DNAs are in very few cells and cannot increase further because of lack of replication. *Trypanosoma brucei*, a parasite related to *Leishmania*, has a much stricter replication system where episomal vector maintenance is the exception [45], and no gene amplification has been described in this parasite, except for changes in ploidy [46]. We searched for the presence of repeated sequences in *T. brucei* and found 773 DRs or IRs that could potentially lead to 1,848 amplifications. We tested the rearrangements at the level of selected repeats and obtained PCR products (Figure S8) whose sequencing confirmed the predicted rearrangements. Thus, *T. brucei* has repeated sequences where gene rearrangements can occur but further amplification is not observed, possibly because these extrachromosomal DNAs cannot replicate. Additional organisms need to be tested for the presence of repeated sequences and for DNA rearrangements by sensitive PCR. Although this may not be as adaptive and flexible as in *Leishmania*, it may provide transient selective advantage to a population. It is salient to point out that long repeated sequences were found in large plasmids of *Rhizobium* and rearrangements mediated by HR at the level of the repeats were evidenced by a PCR-based method similar to the one presented here [47].

*Leishmania* is known to have extensive plasticity in its genome. Previous studies revealed that *Leishmania* chromosomes can be aneuploid, being either monosomic, disomic, or polysomic [28],[29],[48],[49]. Evidence for mosaic aneuploidy was also provided in single cells by FISH analysis [50]. In a population, the average copies of a chromosome may be two, but individual cells may have one, two, or three copies of one specific chromosome [50]. Here, we provide an additional layer of complexity and show an extreme example of genome plasticity. Cells in the population have a common core genome, but single cells differ from the rest of the population by carrying one or several circular and/or linear extrachromosomal amplicons. Individual cells differ in their amplicon complement, and upon selection with either drugs (Figures 4, 7, and 8) or culture conditions (Figure 6), a subpopulation would emerge where the amplicon copy number per cell would increase and then expand to become a majority of the population (Figure 5). Extrachromosomal elements have been found in field isolates [23],[49],[51], suggesting that this is not only found in cells cultured *in vitro*.

The phenomenon of DNA amplification is reversible, as removal of the selective pressure leads to the population reverting to equilibrium (Figures 4 and 6). Whole genome rearrangements is thus a highly dynamic process that continuously takes place in *Leishmania*. We propose that this plasticity is used by *Leishmania* as one strategy to respond to its changing environment. Indeed, within the whole population, a subpopulation having a specific amplicon may be sufficient to provide the necessary advantage to allow the population to thrive. Subpopulations with amplified DNAs thrive during selective conditions and can help (possibly by secreting bioactive molecules or detoxifying the milieu) remaining cells of the population that lack the specific locus amplified. Our assay of locus deletion revealed that this type of rearrangement seems to be as frequent as those leading to extrachromosomal elements (Figure 3). The cost of deleting several genes between repeats may be high, but it is possible that within a population, a subpopulation with genomic deletion (e.g., a drug transporter) may help the population to grow in a selective environment, and upon removal of the selection, these specific cells would be lost from the population.

*Leishmania* is an early divergent eukaryote that does not regulate gene expression at the level of transcription initiation. Undoubtedly, there are several layers of regulation including posttranscriptional and translational mechanisms [13]. Along with chromosome aneuploidy [28],[49],[50], stochastic gene rearrangement is a strategy that *Leishmania* evolved to respond to its environment. At the population level, we have seen that most regions bordered by repeats are rearranged and subpopulations of these cells can be selected. The mechanisms leading to circular and linear amplicons are likely to differ (Figure 1). Indeed, RAD51 is important for the formation of circular amplicons but not linear ones (Figure 8). RAD51 is not essential, however, as two out of seven *RAD51* mutants selected for arsenite resistance had circular amplicons (Figure 8C, lanes 2 and 7), suggesting that other recombinases are involved. As the *Leishmania* genome displays high plasticity, the targeting of enzymes involved in gene rearrangements may prevent the parasite from adapting to drugs and hinder the emergence of the most prevalent resistance mechanisms. This is in line with the concept of targeting components of the mutagenesis pathways leading to adaptive mutations in response to stressful environments (reviewed in [52]). The use of such inhibitors (also known as anti-evolvability drugs) would represent novel therapeutic strategies for preventing the evolution of antimicrobial resistance [52]–[54].

## Materials and Methods

### Bioinformatic Analyses and Primer Design

Genome sequences and annotations were obtained from GeneDB in XML or Artemis format. Analyses were performed on the genome sequence of *L. major* Friedlin version 5.2, *L. infantum* JPCM5 version 3, and *L. braziliensis* clone M2904 version 2. Intergenic sequences were extracted and blasted against their respective chromosome. Blast hits were filtered for identities higher than 85% and lengths between 200 and 2,500 nucleotides. Redundant hits were removed, and repeated sequences were given unique identifiers. For each chromosome, repeated sequences were clustered by sequence homology. Repeated regions were screened for SIDERs as described [37]. Primers were designed for all putative recombination events using Primer3 and designed within 150 nucleotides from the repeated sequence with the orientations shown in Figure 1A and 1B. Optimal primer length was 23 nucleotides, and optimal Tm was 58°C. Input files for Primer 3 were created using in-house perl scripts. A list of the primers used can be found in Table S5.

### Pulse-Field Gel Electrophoresis and Southern Blot Hybridization

Intact chromosomes were prepared from late log phase cultures of *Leishmania* promastigotes and separated by pulse-field gel electrophoresis using a Bio-Rad CHEF-DR III apparatus at 5 V/cm and 120° separation angle as described [39]. Gels were transferred and hybridized with [α-^32^P]dCTP-labeled DNA probes according to standard protocols.

### DNA Preparation for Sensitive PCR Assays

Late log phase promastigotes (15–20 ml) were pelleted at 3,000 rpm for 5 min, and pellets were washed with HEPES-NaCl, resuspended in suspension buffer (100 mM EDTA, 100 mM NaCl, 10 mM TRIS pH 8.0), and lysed in 1% SDS with 50 µg/ml proteinase K for 2 h at 37°C. The DNA was extracted with 1 volume of phenol, precipitated with 2 volumes of 99% ethanol, washed with 70% ethanol twice, and dissolved in 1 ml TE. RNAse (20 µg/ml) was added and incubated for 30 min at 37°C, followed by addition of 50 µg/ml proteinase K and 0.1% SDS at 37°C for 30 min. DNA was extracted with 1 volume of phenol, precipitated, and washed as above, then dissolved in MilliQ water. DNA was quantified using a Nanodrop spectrophotometer.

### Semiquantitative Polymerase Chain Reaction and Estimate of the Rearrangement Frequency

The PCR products, which needed to be longer than the size of the repeats used for recombination, required optimizations. PCR reaction mixture consisted of 100 ng of phenol-purified genomic DNA isolated as described above, 4 µM of forward and reverse primers, 2 mM dNTPs, 1.25 U of FastStart Taq DNA polymerase (Roche), 1×PCR buffer + MgCl_2_, and 3.33 mg/ml BSA. The total reaction mixture was made up to 25 µl by addition of the genomic DNA. Each PCR reaction was standardized as follows: an initial denaturation at 94°C for 4 min, denaturation at 95°C for 15 s, annealing for 30 s, elongation at 72°C for 1 min, and a final extension at 72°C for 5 min. For each PCR reaction, the annealing temperature was optimized as well as the number of cycles to prevent saturation of the amplification. The housekeeping gene *GAPDH* was used as a control to normalize the amount of DNA loaded in each reaction. Saturation of band intensities of the amplified PCR products was determined using the AlphaImager 2000 software. We used *L. major MTX60.4* mutant, which contains the *DHFR* amplicon [28], to make a standard curve and determine the frequencies of rearrangement. Prior to DNA extraction, the MTX60.4 cells were mixed with *L. major* wild-type cells from 10^1^- to 10^10^-fold, resulting in the dilution of the amplicon in DNA extracts. The semiquantitative PCR described above revealed a relative decrease in amplification frequency for the dilution 10^−1^ to 10^−6^, consistent with the dilution of the amplicons (Figure S3). Higher dilutions of the targets (10^−7^ to 10^−10^) did not further decrease the signal intensities after PCR quantitation, which suggests that the 10^−6^ dilution represents the basal rearrangement frequency (Figure S3). Considering that MTX60.4 cells have an average of 10 amplicons per cells [28], we estimated the rearrangement frequency at 10^−6^ to 10^−7^ amplicon per cell. We carried out similar dilution experiments with *L. infantum* MTX20.5, which contains both the *DHFR-TS* circular amplicon and the *PTR1* linear amplicon [28], and found similar rearrangement rates of 10^−6^ to 10^−7^. To quantitate the amplicons in the stressed samples, PCRs were done with the same dilutions and optimized cycle numbers as for the standard curve. The same amount of the PCR amplification reactions was loaded on an agarose gel, and the signals were quantified and normalized.

### Colony-Based PCR

Parasites from passages P3, P6, and P8 at 0.5×MTX EC_50_ and from passage P4 at 1×MTX EC_50_ (P4^1^) were plated on SDM agar for 10 d. Single colonies were picked from the plates and independently lysed in 50 µl of InstaGen Matrix solution (BioRad) according to the manufacturer's recommendations. PCR reaction mixture and conditions were the same as described above but with 1 µl of lysed colony-derived parasites instead of 100 ng phenol-purified genomic DNA. The optimized number of cycles for the detection of the novel junction originating from the *DHFR-TS* amplicon was set to 30 cycles. For the unselected parasites (P0 passage), no amplification was observed even when the number of cycles was 40 cycles. The housekeeping gene *GAPDH* was used as a control.

### Generation of a *L. infantum RAD51* Null Mutant

The first allele of *LinJ.28.0580* (*RAD51*) of *L. infantum* was deleted by HR using the noncoding flanking regions of the gene fused to the marker neomycin phosphotransferase (*NEO*). The deletion of the second allele could not be obtained by HR using the same flanking regions. An insertional inactivation strategy was performed where the hygromycin phosphotransferase gene (*HYG*) was inserted within the ORF. The primers used for inactivation of both alleles are listed in Table S5. Transfectants were selected in the presence of 40 µg/ml of G418 (Geneticin) and 300 µg/ml of hygromycin B, and integrations were confirmed by Southern blots and PCR.

### Recombination Assays

Recombination assay of 5′*GSH1*-*BLA*-3′ GSH1 was measured as described [55]. Briefly, 5 µg of the blasticidin (BLA) inactivation construct was transfected in 4×10^7^ WT or *RAD51* null mutant cells. After 24 h, cells were preselected with 40 µg/ml blasticidin, and after another 24 h, 5–7×10^6^ cells were plated in the presence of 50 µg/ml blasticidin (Invitrogen) in triplicates and the number of colonies per plate was counted after 10–15 d.

### NGS

Library was prepared using the Nextera library preparation kit (Illumina, Carlsbad, CA) following the manufacturer's protocol. Library was quantified with Picogreen, and 20 µl of 2.5 nM diluted DNA was used for library preparation. Library was denatured and diluted to 20 pM following the protocol recommended for sequencing of Nextera libraries on the MiSeq sequencer (Illumina). Sequencing was performed on the MiSeq system using paired-end reads of 150 nucleotides. Genomes were assembled using Ray 2.0.0 [56]. Reads were aligned to *L. infantum* JPCM5 version 4 using the bwa aligner [57], and sequencing coverage was assessed for each position of chromosome 6. The sequencing data for the FCPO amplicon are available at the EMBL European Nucleotide Archive (http://www.ebi.ac.uk/ena) under the study accession number ERP002431, sample accession ERS227354.

## Supporting Information

Figure S1 Distance between repeated sequences within the same chromosome for (A) DRs and (B) IRs. The abscissa is a log10 scale of kbp, and the *y*-axis corresponds to the number of couples of repeats in the range defined on the abscissa. Most of the couples of DRs and IRs are 1 to 100 kbp distant. Distribution of IRs (C) and DRs (D) along the chromosomes. IRs are enriched in regions near the telomeres, whereas DRs are more uniformly dispersed. The abscissa corresponds to the distance between the repeats and the closest telomere end, and the *y* axis to the number of couples in the range defined on the abscissa.
(TIF)

Figure S2 Amplicon detection in intracellular *Leishmania*. J774 murine macrophage cells were infected with *L. infantum* (MHOM/MA/67/ITMAP-263) promastigotes at a parasite/macrophage ratio of 10∶1 for 3 h. Noninternalized parasites were removed by several washes. After 4 d, the cells were collected, washed with HEPES-NaCl, and homogenized in resuspension buffer (100 mM EDTA, 100 mM NaCl, 10 mM TRIS pH 8.0). The cells were lysed and DNA extracted as described in Materials and Methods. PCR reaction mixture for detection of the PTR1 circular and linear amplicons consisted of 500 ng of the prepared genomic DNA, and sensitive PCR was carried out as described under Materials and Methods. The housekeeping chromosomal gene glyceraldehyde-3-phosphate dehydrogenase (GAPDH) was used as an internal control. All amplicons were sequenced to confirm their identity. Lane 1, intracellular amastigotes; lane 2, no template control.
(TIF)

Figure S3 Determination of frequency of gene rearrangements in *Leishmania*. *L. major MTX60.4* cells were diluted 10^1^- to 10^10^-fold with *L. major* LV39 wild-type cells, and PCR to detect DHFR amplicons was conducted. PCR products were loaded on a 1% agarose gel (A) and quantified. The chromosomal locus GAPDH was used for normalization. The semiquantitative PCRs were realized with an increasing number of cycles until a clear PCR product was detected for the wild-type strain. The dilutions 10^−8^ to 10^−10^ gave the same amplification rate than the wild-type strain, indicating that those samples contained the same number of amplicons. Because the MTX60.4 cells contain at least 10 amplicons per cell (Ubeda et al., 2008) [28], these data indicate that the rate of the *DHFR* genomic rearrangements is higher than 10^−7^. The PCR quantitation data at cycle 25 were used to draw a standard curve (B), which was then used to determine, after 25 PCR cycles, the quantity of amplicons in the MTX stressed population.
(TIF)

Figure S4 Selection for MTX resistance and *DHFR-TS* amplification in *L. major*. (A) Selection scheme of *L. major* for eight continuous passages at 0.5× MTX EC_50_ or by 2-fold increments from 0.5× MTX EC_50_ up to 8× MTX EC_50_. DNAs extracted at selected passages were further analyzed by PCR and Southern blots for the presence of the *DHFR-TS* amplicon in Figure 5 (*) and Figure S4C (^+^). P1–P8 refers to the number of passages performed at each MTX concentration. (B) Map of the *DHFR-TS* locus and its amplified region. The 10 kb EcoRV–BglII chromosomal fragment and the 4 kb BglII–BglII rearranged fragment diagnostic of amplification are indicated. Black arrows represent the DRs involved in HR. The probe used for differentiating the chromosomal and amplified loci by Southern blots corresponds to the first 1,000 bp of the gene *LinJ.06.0830* and is indicated by the line under the map. (C) Southern blots of DNAs isolated from *L. major* selected step-by-step for MTX resistance by 2-fold MTX increments until they grew at 8× EC_50_ (these DNAs were extracted from passages with a ‘^+^’ sign in Figure S4A). The blot was hybridized to the probe indicated in Figure S4B, allowing the discrimination of the rearranged and chromosomal bands. The blot was rehybridized with a probe covering the coding sequence of *DHFR-TS* (*DHFR*) and to a *GAPDH* probe to monitor the DNA loaded in each lane. Fold increases were normalized with the *GAPDH* signal.
(TIF)

Figure S5 *L. infantum RAD51* null mutant and associated phenotypes. (A) Diagram of the *RAD51* locus in *L. infantum* WT, and with the *NEO* deletion and *HYG* disruption cassettes, with localization of primers a–f and size of the PCR products. (B) PCR fragments with primers a–f confirming inactivation cassettes insertion in the *RAD51* locus. Molecular weights are indicated on the left, and the various alleles are pinpointed on the right. Lane 1, *L. infantum* WT; lane 2, *RAD51/RAD51::NEO*; lane 3, *RAD51::NEO/RAD51::HYG*. White lines indicate nonadjacent lanes that have been brought together for producing the figures. (C) Effect of methylmethane sulphonate (MMS) on cell growth. *L. infantum* WT PSPα*ZEO*α (filled triangle), *RAD51* ^−/−^ PSPα*ZEO*α (filled square), and *RAD51* ^−/−^ PSPα*ZEO*α*RAD51* (filled circle) were passaged in various concentrations of MMS for 3 d, after which the growth was monitored at 600 nm. Average of three independent experiments is shown. (D) Transformation efficiency of *RAD51* ^−/−^ mutants. *L. infantum* WT PSPα*ZEO*α, *RAD51* ^−/−^PSPα*ZEO*α, and *RAD51* ^−/−^PSPα*ZEO*α*RAD51* were transfected with a linear DNA fragment containing a selectable marker (BLA) flanked by the 5′ and 3′ *GSH1* flanking sequences. Transformation efficiency was calculated by plating an equal number of cells in triplicate on plates containing the selection drug and counting the number of colonies per plate. The graph represents triplicate from two independent experiments.
(TIF)

Figure S6 *MRPA* gene amplification in selected arsenite-resistant *L. infantum* mutants. The DNAs of *L. infantum* WT cells and of the arsenite-resistant mutants (Figure 8B, lanes 7, 8, and 10) were isolated and digested with NcoI and hybridized with *MRPA* and *GAPDH* probes. Dilution experiments with the DNA of As250.8 have indicated that the copy number of the MRPA amplicon is higher than 20 copies in this cell line.
(TIF)

Figure S7 Identification of the *MRPA* locus rearrangements in the SbIII-resistant clones by PCR. (A) The map of the *MRPA* locus is shown with the repeats A–D and the primers 1–9. (B) *L. infantum* WT cells (left panel), *RAD51* null mutant (middle panel), and *RAD51* revertant cells (right panel) were selected for arsenite resistance, and all the arsenite-resistant clones selected independently displayed *MRPA* amplifications (Figure 8). By PCR, we determined the rearrangements that occurred using specific primer pairs for each couple of repeats. Fewer efforts were made for having sensitive PCR as we were dealing with highly amplified DNAs.
(TIF)

Figure S8 Evidence for gene rearrangements at the level of DRs and IRs in *Trypanosoma brucei*. A bioinformatics analysis revealed at least 773 intergenic repeated sequences in *T. brucei*. Four examples are shown here. Evidence for rearrangements at the level of these repeats was obtained by PCR using appropriate primers. PCR products of the right size were obtained for each reaction and sequenced. P, PCR product; M, molecular weight marker. White lines indicate nonadjacent lanes that have been brought together for producing the figure.
(TIF)

Table S1 *L. major* pairs of repeated sequences with putative gene amplification products.
(XLSX)

Table S2 Properties of *L. major* RAGs.
(XLSX)

Table S3 *L. infantum* pairs of repeated sequences with putative gene amplification products.
(XLSX)

Table S4 *Leishmania braziliensis* pairs of repeated sequences with putative gene amplification products.
(XLSX)

Table S5 PCR primers used in this study.
(XLSX)

## Abbreviations

CNV: copy number variation
DRs: direct repeated sequences
FBS: fetal bovine serum
HR: homologous recombination
IRs: inverted repeats
MTX: methotrexate
RAG: repeat alignment group
SIDERs: short interspersed degenerate retroposons
