# Supplementary figures and images for: Genome-Wide Stochastic Adaptive DNA Amplification at Direct and Inverted DNA Repeats in the Parasite Leishmania
Source: PLoS Biol. 2014 May 20;12(5):e1001868. doi: 10.1371/journal.pbio.1001868 (PMC4028189; doi:10.1371/journal.pbio.1001868)

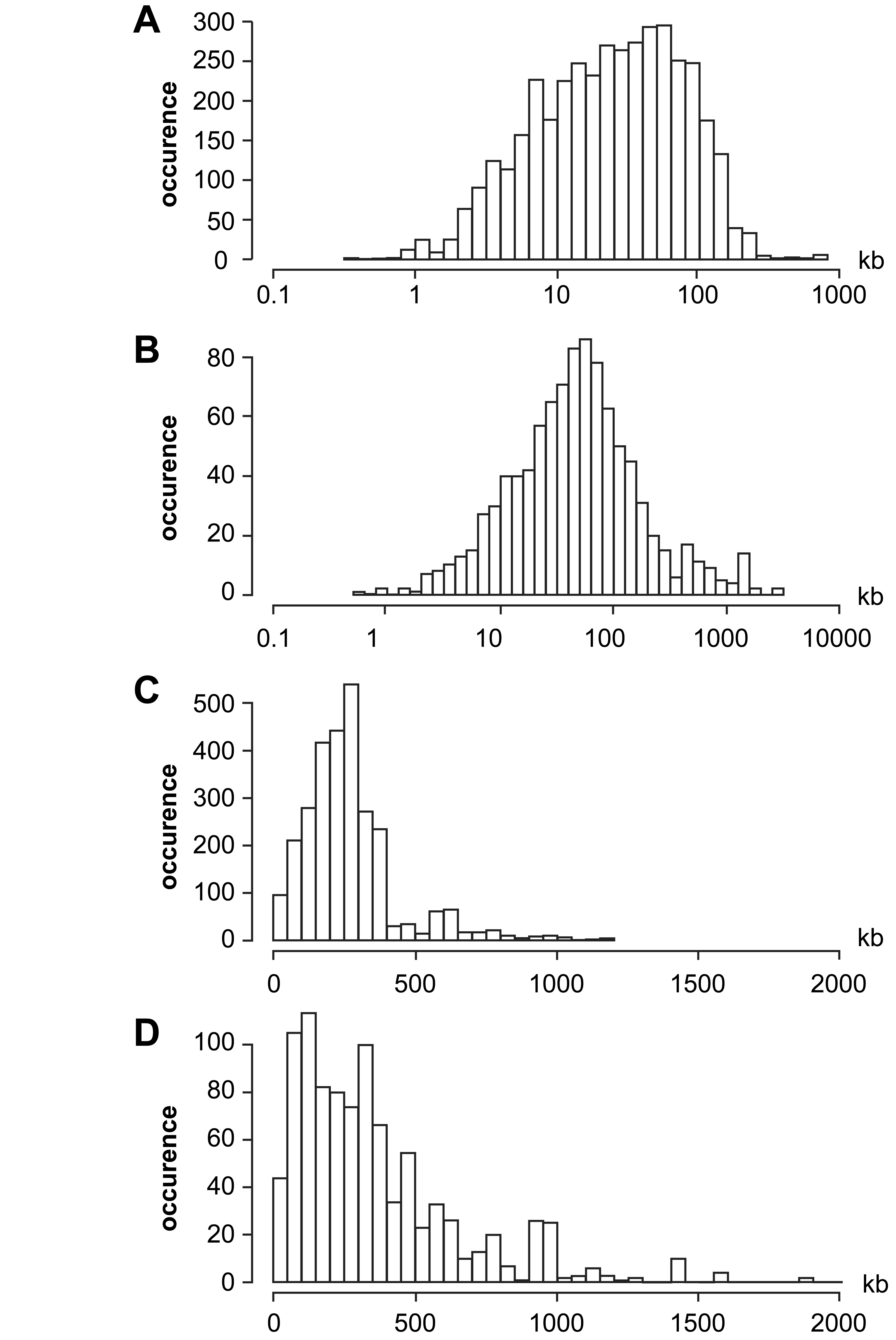

Supplement: Figure S1 — Distance between repeated sequences within the same chromosome for (A) DRs and (B) IRs. The abscissa is a log10 scale of kbp, and the y-axis corresponds to the number of couples of repeats in the range defined on the abscissa. Most of the couples of DRs and IRs are 1 to 100 kbp distant. Distribution of IRs (C) and DRs (D) along the chromosomes. IRs are enriched in regions near the telomeres, whereas DRs are more uniformly dispersed. The abscissa corresponds to the distance between the repeats and the closest telomere end, and the y axis to the number of couples in the range defined on the abscissa. (TIF) [file pbio.1001868.s001.tif]

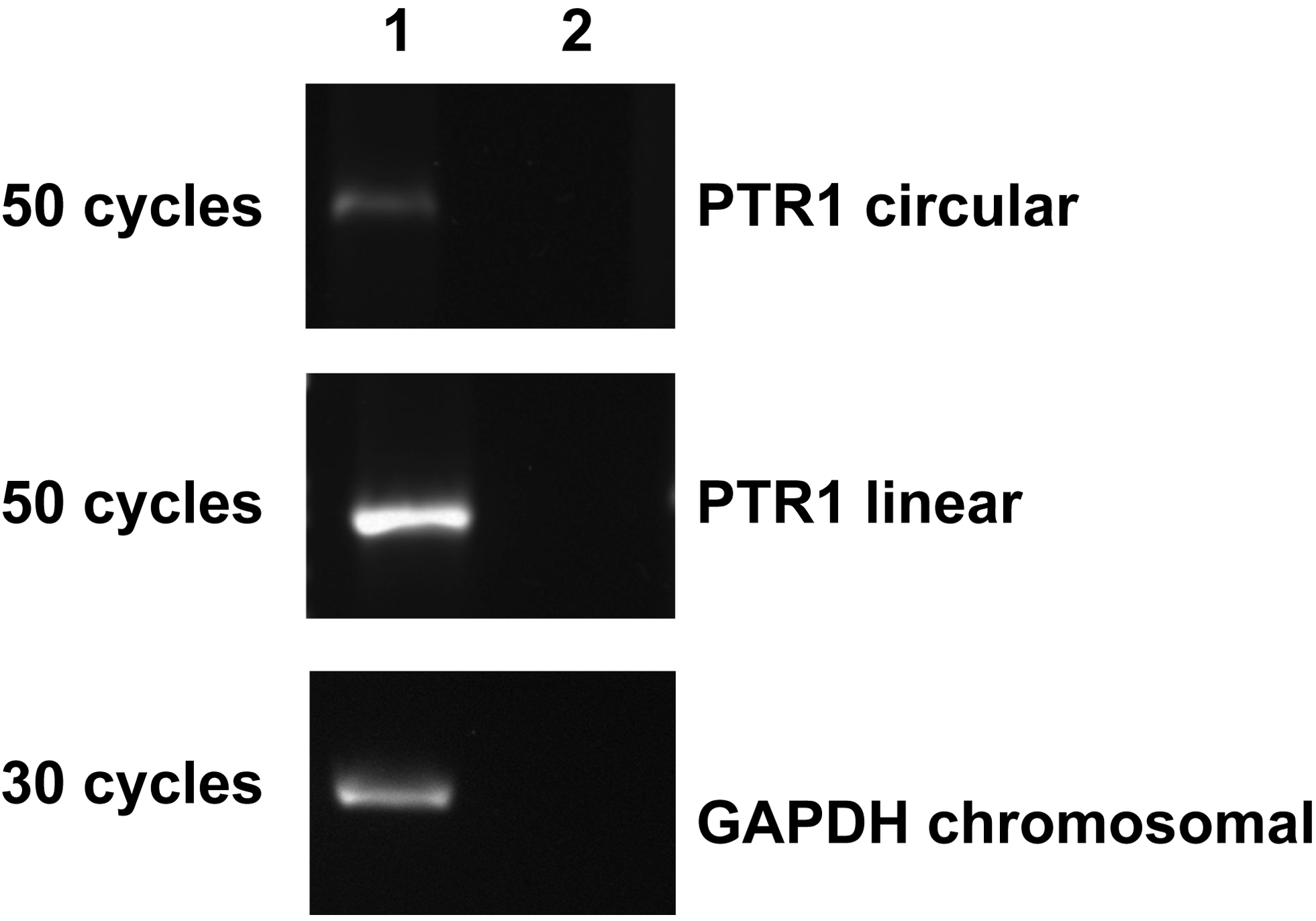

Supplement: Figure S2 — Amplicon detection in intracellular Leishmania. J774 murine macrophage cells were infected with L. infantum (MHOM/MA/67/ITMAP-263) promastigotes at a parasite/macrophage ratio of 10∶1 for 3 h. Noninternalized parasites were removed by several washes. After 4 d, the cells were collected, washed with HEPES-NaCl, and homogenized in resuspension buffer (100 mM EDTA, 100 mM NaCl, 10 mM TRIS pH 8.0). The cells were lysed and DNA extracted as described in Materials and Methods. PCR reaction mixture for detection of the PTR1 circular and linear amplicons consisted of 500 ng of the prepared genomic DNA, and sensitive PCR was carried out as described under Materials and Methods. The housekeeping chromosomal gene glyceraldehyde-3-phosphate dehydrogenase (GAPDH) was used as an internal control. All amplicons were sequenced to confirm their identity. Lane 1, intracellular amastigotes; lane 2, no template control. (TIF) [file pbio.1001868.s002.tif]

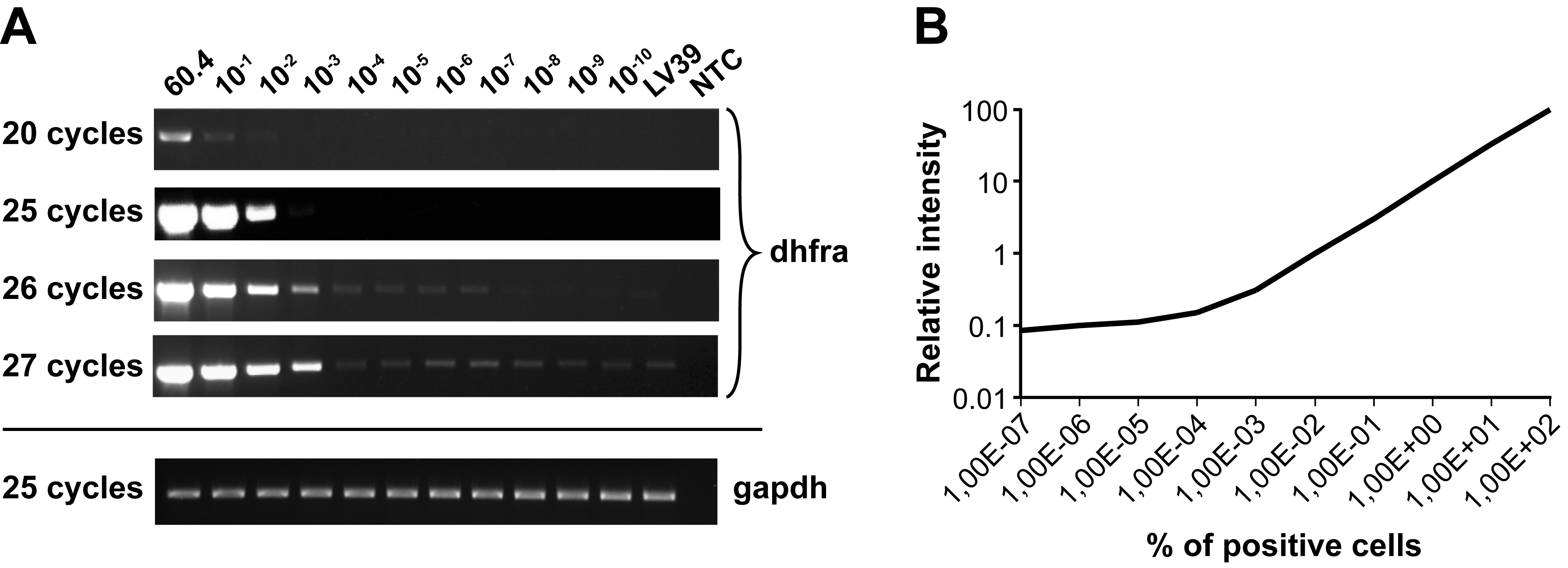

Supplement: Figure S3 — Determination of frequency of gene rearrangements in Leishmania. L. major MTX60.4 cells were diluted 101- to 1010-fold with L. major LV39 wild-type cells, and PCR to detect DHFR amplicons was conducted. PCR products were loaded on a 1% agarose gel (A) and quantified. The chromosomal locus GAPDH was used for normalization. The semiquantitative PCRs were realized with an increasing number of cycles until a clear PCR product was detected for the wild-type strain. The dilutions 10−8 to 10−10 gave the same amplification rate than the wild-type strain, indicating that those samples contained the same number of amplicons. Because the MTX60.4 cells contain at least 10 amplicons per cell (Ubeda et al., 2008) [28], these data indicate that the rate of the DHFR genomic rearrangements is higher than 10−7. The PCR quantitation data at cycle 25 were used to draw a standard curve (B), which was then used to determine, after 25 PCR cycles, the quantity of amplicons in the MTX stressed population. (TIF) [file pbio.1001868.s003.tif]

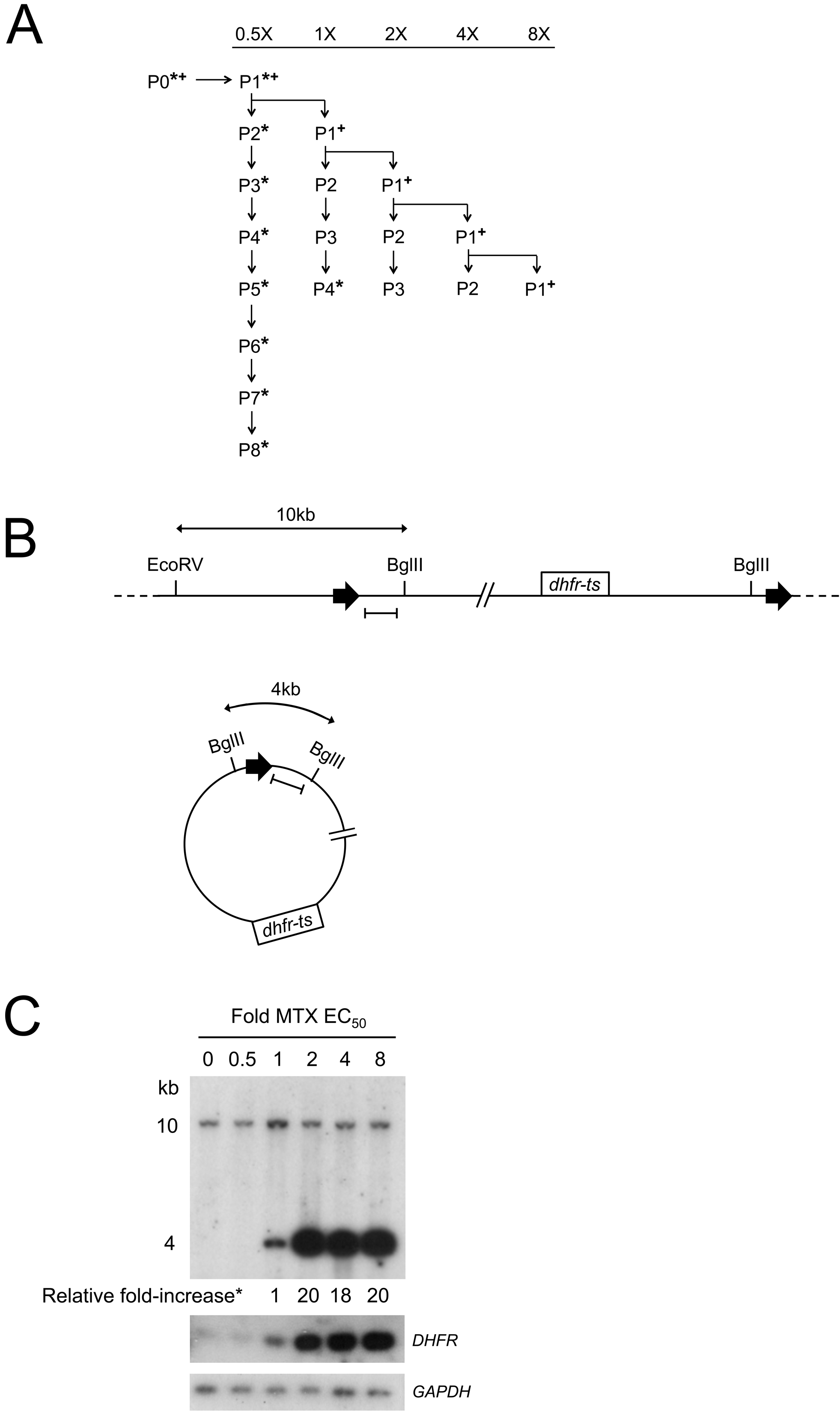

Supplement: Figure S4 — Selection for MTX resistance and DHFR-TS amplification in L. major. (A) Selection scheme of L. major for eight continuous passages at 0.5× MTX EC50 or by 2-fold increments from 0.5× MTX EC50 up to 8× MTX EC50. DNAs extracted at selected passages were further analyzed by PCR and Southern blots for the presence of the DHFR-TS amplicon in Figure 5 (*) and Figure S4C (+). P1–P8 refers to the number of passages performed at each MTX concentration. (B) Map of the DHFR-TS locus and its amplified region. The 10 kb EcoRV–BglII chromosomal fragment and the 4 kb BglII–BglII rearranged fragment diagnostic of amplification are indicated. Black arrows represent the DRs involved in HR. The probe used for differentiating the chromosomal and amplified loci by Southern blots corresponds to the first 1,000 bp of the gene LinJ.06.0830 and is indicated by the line under the map. (C) Southern blots of DNAs isolated from L. major selected step-by-step for MTX resistance by 2-fold MTX increments until they grew at 8× EC50 (these DNAs were extracted from passages with a ‘+’ sign in Figure S4A). The blot was hybridized to the probe indicated in Figure S4B, allowing the discrimination of the rearranged and chromosomal bands. The blot was rehybridized with a probe covering the coding sequence of DHFR-TS (DHFR) and to a GAPDH probe to monitor the DNA loaded in each lane. Fold increases were normalized with the GAPDH signal. (TIF) [file pbio.1001868.s004.tif]

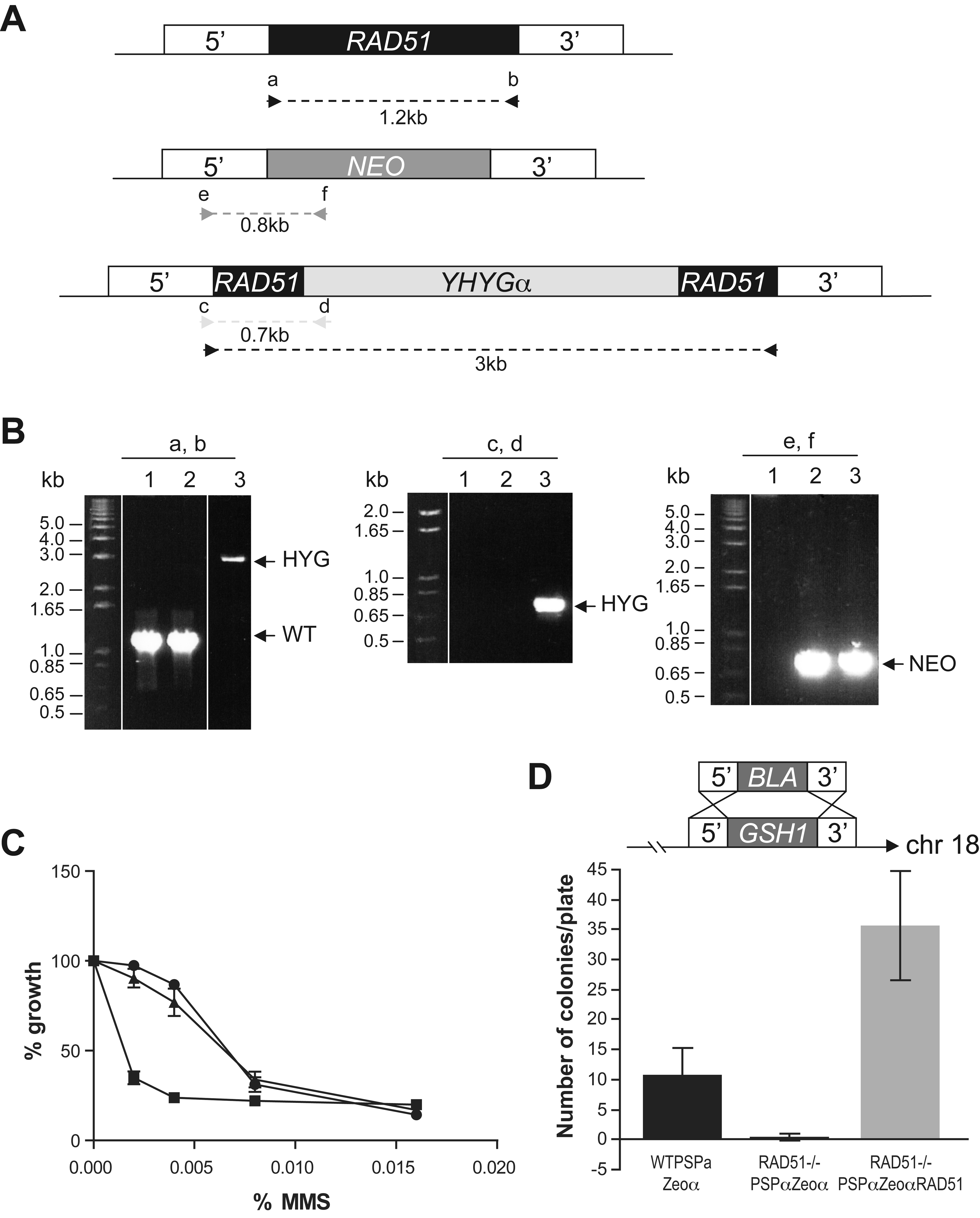

Supplement: Figure S5 — L. infantum RAD51 null mutant and associated phenotypes. (A) Diagram of the RAD51 locus in L. infantum WT, and with the NEO deletion and HYG disruption cassettes, with localization of primers a–f and size of the PCR products. (B) PCR fragments with primers a–f confirming inactivation cassettes insertion in the RAD51 locus. Molecular weights are indicated on the left, and the various alleles are pinpointed on the right. Lane 1, L. infantum WT; lane 2, RAD51/RAD51::NEO; lane 3, RAD51::NEO/RAD51::HYG. White lines indicate nonadjacent lanes that have been brought together for producing the figures. (C) Effect of methylmethane sulphonate (MMS) on cell growth. L. infantum WT PSPαZEOα (filled triangle), RAD51 −/− PSPαZEOα (filled square), and RAD51 −/− PSPαZEOαRAD51 (filled circle) were passaged in various concentrations of MMS for 3 d, after which the growth was monitored at 600 nm. Average of three independent experiments is shown. (D) Transformation efficiency of RAD51 −/− mutants. L. infantum WT PSPαZEOα, RAD51 −/−PSPαZEOα, and RAD51 −/−PSPαZEOαRAD51 were transfected with a linear DNA fragment containing a selectable marker (BLA) flanked by the 5′ and 3′ GSH1 flanking sequences. Transformation efficiency was calculated by plating an equal number of cells in triplicate on plates containing the selection drug and counting the number of colonies per plate. The graph represents triplicate from two independent experiments. (TIF) [file pbio.1001868.s005.tif]

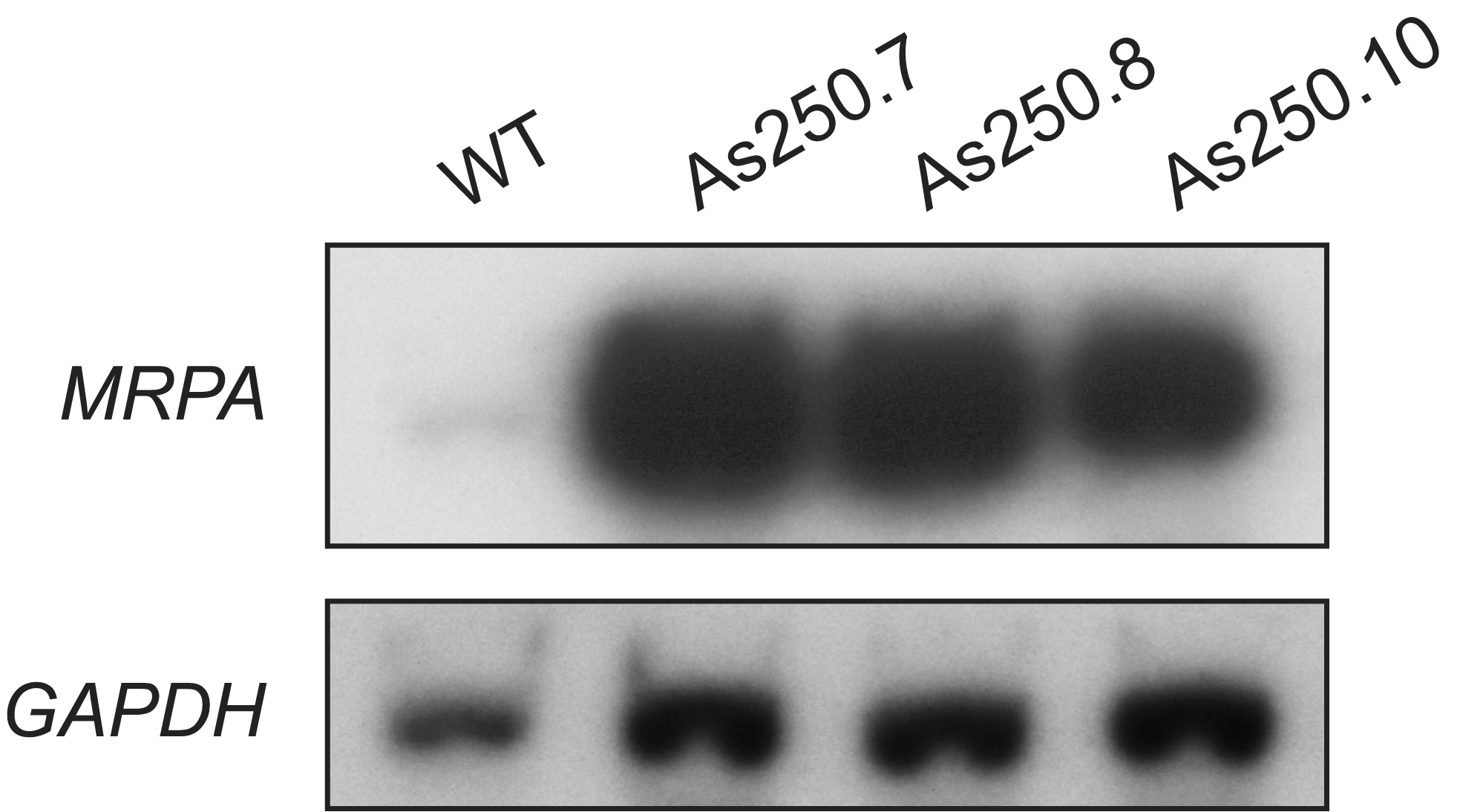

Supplement: Figure S6 — MRPA gene amplification in selected arsenite-resistant L. infantum mutants. The DNAs of L. infantum WT cells and of the arsenite-resistant mutants (Figure 8B, lanes 7, 8, and 10) were isolated and digested with NcoI and hybridized with MRPA and GAPDH probes. Dilution experiments with the DNA of As250.8 have indicated that the copy number of the MRPA amplicon is higher than 20 copies in this cell line. (TIF) [file pbio.1001868.s006.tif]

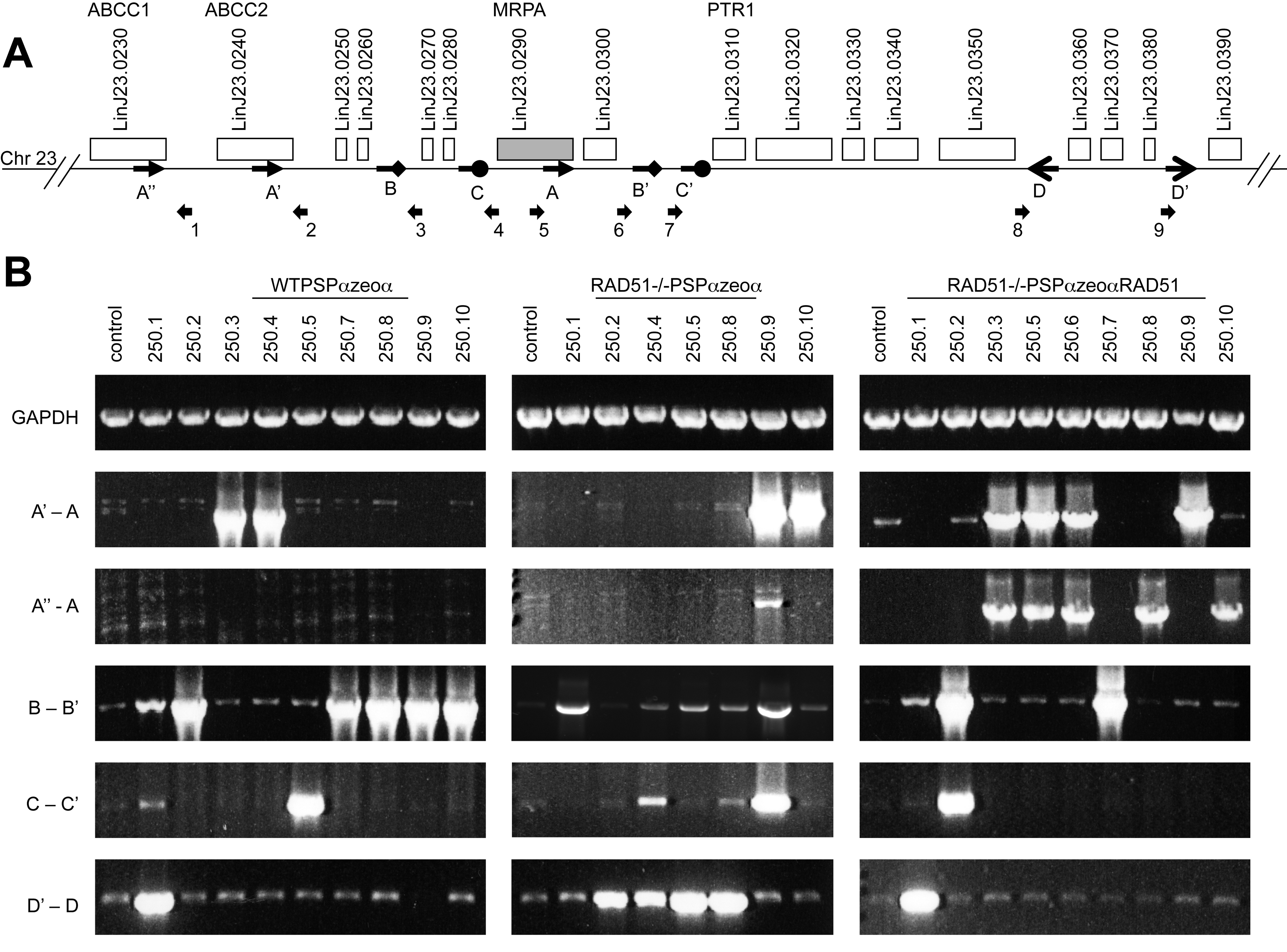

Supplement: Figure S7 — Identification of the MRPA locus rearrangements in the SbIII-resistant clones by PCR. (A) The map of the MRPA locus is shown with the repeats A–D and the primers 1–9. (B) L. infantum WT cells (left panel), RAD51 null mutant (middle panel), and RAD51 revertant cells (right panel) were selected for arsenite resistance, and all the arsenite-resistant clones selected independently displayed MRPA amplifications (Figure 8). By PCR, we determined the rearrangements that occurred using specific primer pairs for each couple of repeats. Fewer efforts were made for having sensitive PCR as we were dealing with highly amplified DNAs. (TIF) [file pbio.1001868.s007.tif]

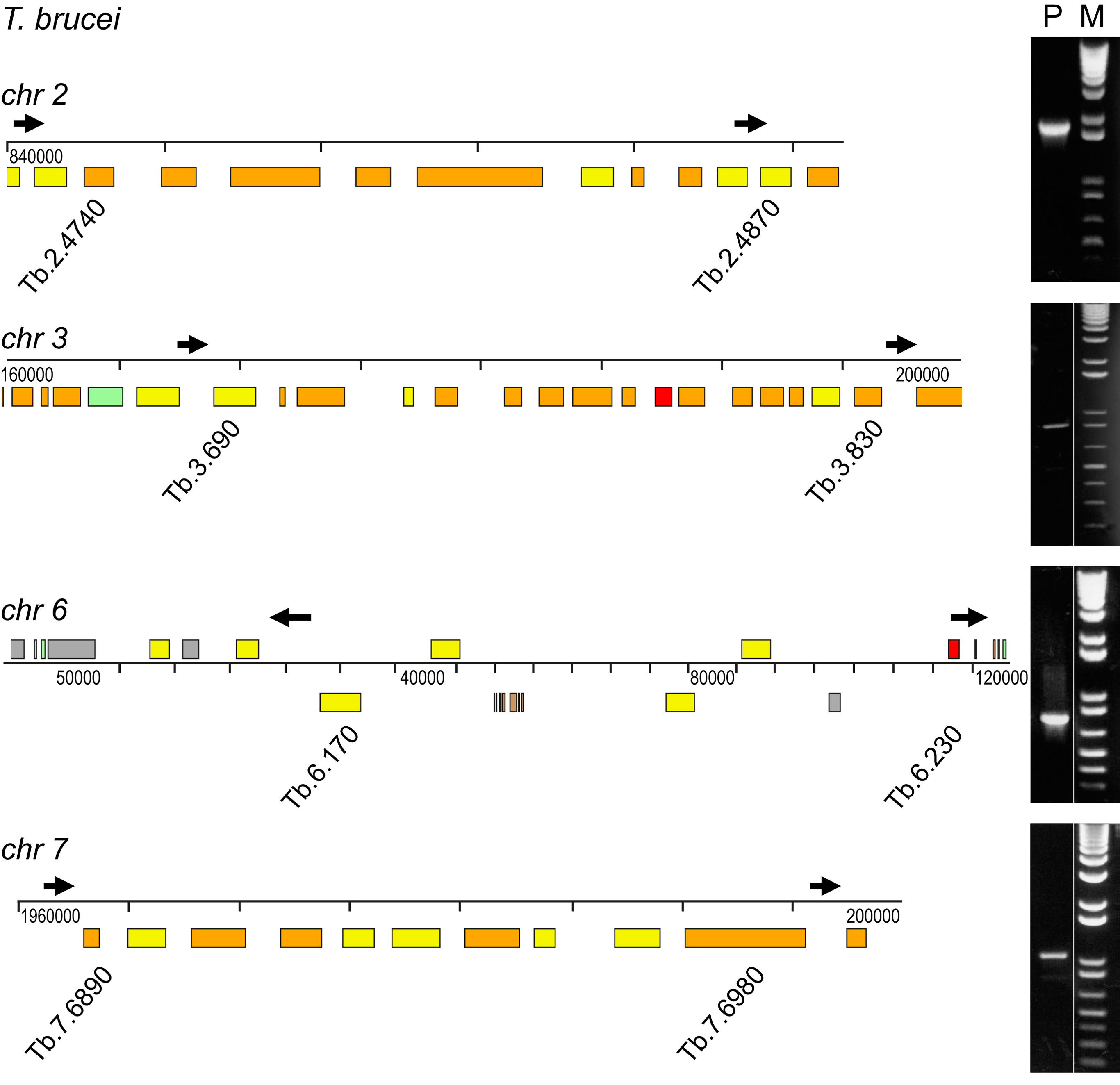

Supplement: Figure S8 — Evidence for gene rearrangements at the level of DRs and IRs in Trypanosoma brucei. A bioinformatics analysis revealed at least 773 intergenic repeated sequences in T. brucei. Four examples are shown here. Evidence for rearrangements at the level of these repeats was obtained by PCR using appropriate primers. PCR products of the right size were obtained for each reaction and sequenced. P, PCR product; M, molecular weight marker. White lines indicate nonadjacent lanes that have been brought together for producing the figure. (TIF) [file pbio.1001868.s008.tif]
